# Ovoid cell is an inducible small-sized morphotype that enhances proliferation and antifungal drug tolerance in the human fungal pathogen *Cryptococcus neoformans*

**DOI:** 10.1371/journal.ppat.1014302

**Published:** 2026-06-17

**Authors:** Xitong Zhang, Xiaoji Shu, Ya Liu, Yujia Zhao, Hui Chen, Fan Xiong, Mei Kang, Chengjun Cao

**Affiliations:** 1 College of Pharmaceutical Sciences, Southwest University, Chongqing, China; 2 Department of Laboratory Medicine, Clinical Laboratory Medicine Research Center, West China Hospital, Sichuan University, Sichuan Clinical Research Center for Laboratory Medicine, Sichuan, China; Virginia Tech, UNITED STATES OF AMERICA

## Abstract

*Cryptococcus neoformans* is the leading cause of fungal meningoencephalitis. Cellular heterogeneity during cryptococcal infection contributes to host adaptation and fungal pathogenesis. *C. neoformans* titan cells and seed cells represent enlarged and small-sized morphotypes, respectively, which exhibit distinct transcriptional profiles and can be induced by environmental factors. In this work, we describe a distinct small morphotype of *C. neoformans*, referred to as ovoid cells. The formation of ovoid cells is promoted by host-related conditions such as nutrient limitation and elevated CO_2_ levels, which was observed during the late stage of cryptococcal infection. In addition to their smaller size compared to typical yeast cells, ovoid cells highly express *OSP1*, a marker distinguishes ovoid cells from other small morphotypes, including seed cells and titanides. These cells exhibit an increased budding and proliferation rate, which is consistent with transcriptome data that ovoid cells upregulate cell cycle related genes. We further demonstrate that the glucose repression signaling pathway and the cAMP/PKA pathway are involved in ovoid cell formation in *C. neoformans*. Ovoid cells show reduced fungal virulence but enhanced tolerance under long-term fluconazole treatment, indicating their important role in the balancing virulence and antifungal tolerance within *C. neoformans* populations.

## Author summary

The cell is the fundamental unit of living organisms and cell size control is a universal feature of cellular organisms in nature. *Cryptococcus neoformans*, a major human fungal pathogen, undergoes cell size shift in response to adverse host conditions and antifungal drug treatments. Enlarged titan cells play a crucial role in disease establishment, whereas small-sized cells predominate during the late stage of infection. The development of *in vitro* conditions for titan cell production has advanced our understanding of this large-cell population in *C. neoformans*. Several forms of small cells, including micro-cells, irregular cells, drop cells, titanides, and seed cells, have been described in *C. neoformans*. Seed cells facilitate fungal dissemination to extrapulmonary organs and can be induced by phosphate. However, the environmental triggers and mechanisms underlying the regulation of small-sized *Cryptococcus* cells remain poorly understood. Here, we report a distinct small-sized ovoid cell type of *C. neoformans* and establish a reproducible culture condition for ovoid cells induction. Ovoid cells induced on host relevant conditions exhibit an increased proliferation rate, enhanced tolerance to fluconazole, and attenuated virulence. This study broadens the knowledge of cell size diversity and highlights the role of small cells in the pathogenicity of *C. neoformans*.

## Introduction

Phenotypic plasticity is an important factor enabling fungal adaptation to adverse environmental conditions. Dimorphism, defined as the ability of fungal vegetative cell to exist in either yeast or hyphal forms, provides advantages to numerous fungal pathogens in host environments, including *Candida albicans*, *Blastomyces dermatitidis*, *Histoplasma capsulatum*, and *Ustilago maydis* [1]. For instance, *C. albicans* can undergo yeast-hypha switching during infection, where yeast cells facilitate dissemination and hyphal cells promote tissue invasion [2]. Additionally, within the yeast state, *C. albicans* can transition among white, gray, and opaque forms, which differ in cellular morphology, mating competence, and pathogenicity [3–6]. Other phenotypes, such as GUT cells (gastrointestinal induced transition) and goliath cells, have also been observed in *C. albicans* under specific conditions [7,8]. Polymorphism of *C. albicans* represents a critical virulence attribute during infection. The basidiomycete yeast *Cryptococcus neoformans* was initially regarded as sexual dimorphic that hyphal state exists during mating process and the yeast form predominates during infection [9,10]. However, the observation of cell size heterogeneity (with cell body size ranging from <1 µm to >100 µm) in infected hosts has led to reclassification of *C. neoformans* as a pleomorphic fungus [11].

*Cryptococcus* species, notably *C. neoformans* and *Cryptococcus gattii*, are major causes of fungal meningitis and account for approximately 150,000 deaths annually worldwide [12]. During infection, *Cryptococcus* produces enlarged yeast cells known as titan cells, which are characterized by single large vacuole, thickened cell wall, and polyploidy [13–15]. Titan cells play important roles in establishing and maintaining *Cryptococcus* pathogenicity and conferring resistance to antifungal drugs [13,14,16]. Similar enlarged cellular forms have been observed in other pathogenic fungi, including goliath cells in *C. albicans, Candida dubliniensis*, and *Candida tropicalis* under zinc starvation conditions, as well as giant cells in *Candida auris* following treatment with genotoxic agents [8,17]. However, the contribution of large cells of *Candida* species to fungal pathogenicity remains unclear.

The conditions and signaling pathways regulating large cell formation have been extensively investigated in *C. neoformans* [15,18–21]. Several *in vitro* culture conditions, including minimal medium (MM) [20], titan cell medium (TCM) [19], 10% heat inactivated fetal calf serum (HI-FCS) diluted in phosphate-buffered saline (PBS) [21], serum-free medium [22], human plasma-like medium (HPLM) [23], and Neurobasal (NB) medium [24], have been shown to induce titan-like cell formation in *C. neoformans*. NB and cell culture medium RPMI can also promote titanization of *C. gattii* [24,25]. These *in vitro* titan-like cell inducing conditions have helped identify host-specific environmental factors, such as nutrient limitation, hypoxia, pH, CO_2_, and serum, which contribute to titan cell production [19–21]. Regulatory pathways involving cAMP/protein kinase A signaling, cell cycle and mating pathways and regulators have been identified to be important for titan cell formation [14,19–21,26–30]. Thus, titan cell may represent a unique morphotype specific to *Cryptococcus* species that is relevant to pathogenesis [31].

*C. neoformans* also produces small cells in the lungs and extrapulmonary organs during infection [32–36]. Micro-cells, defined as those with cell body diameter less than 1 μm and thick cell walls, are rarely observed during infection but have been positively correlated with fungal virulence [32,34,37]. Irregular cells occur more frequently in patients receiving antifungal therapy and negatively associated with patient mortality [37]. *C. neoformans* drop cells, observed in the lungs, are dead cells exhibiting distinct morphology with thick cell wall, central vesicle, retracted cytoplasm around vesicle, altered nuclear structure, and metabolic inactivity. The proportion of drop cells varies drastically among isolates and infected hosts [38]. “Seed cell” enhances fungal dissemination and colonization of extrapulmonary organs such as the kidneys and brain [35]. Seed cells exhibit increased mannose exposure and altered surface architecture which enhancing macrophage uptake and organ entry. Seed cell is an inducible morphotype dependent on phosphate acquisition. Various phosphate sources that *C. neoformans* encounters in the environment or during infection facilitate seed cells development [35]. Titanides are cells display very thin cell walls and an oval shape under *in vitro* titan cell inducing conditions [21,39]. Although it remains unknown whether titanides exist during infection or contribute to pathogenicity, their existence highlights the diversity of small cell types in *C. neoformans*.

In this study, we identify a small morphotype in *C. neoformans*, which we designate as ovoid cells. These cells are characterized by their small size (<4 µm), ovoid shape, high proliferation rate, and specific expression of *OSP1*, a marker highly expressed in ovoid cells, but not in seed cells or titanides. We propose that ovoid cell may represent a trade-off between virulence and antifungal drug tolerance, as this population exhibits reduced fungal virulence but improved tolerance under prolonged fluconazole treatment.

## Results

### Small cells predominate in the late stage of *C. neoformans* infection

**Fig 1:**
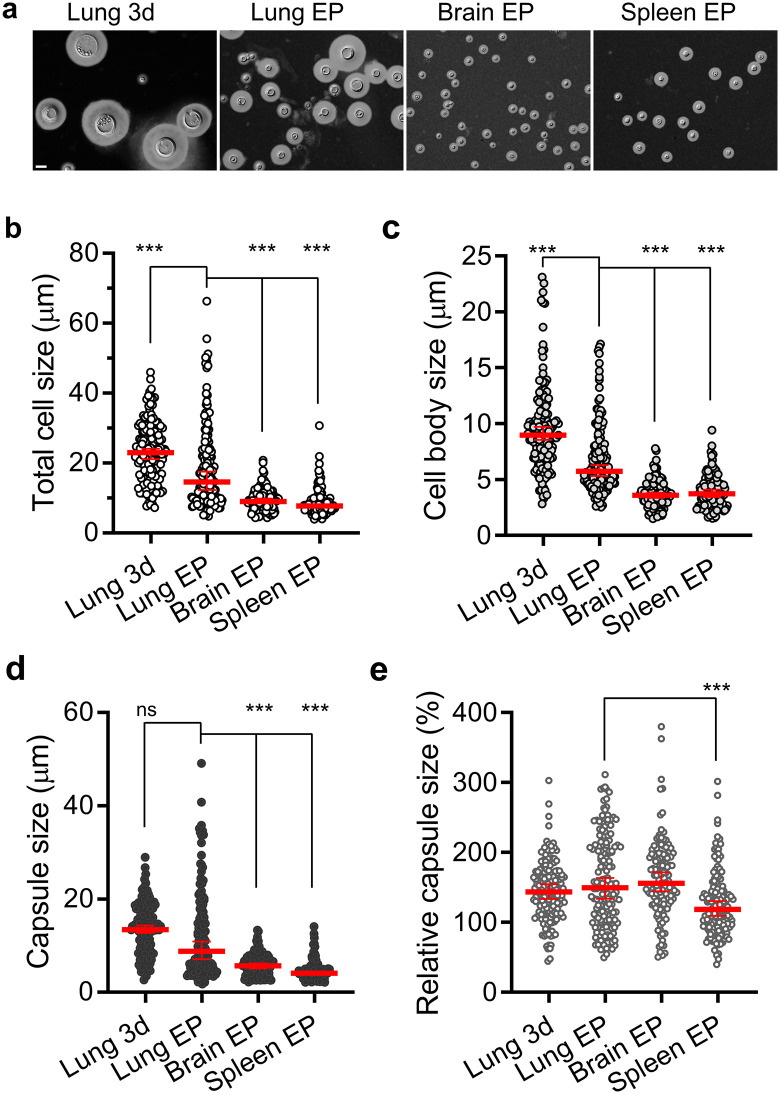
Cell and capsule sizes of *C. neoformans* at the end time point of infection. **(a)** Representative images of *C. neoformans* cells collected from the lungs, brains, and spleens of H99-infected mice after 3 days post-infection (dpi) or at the endpoint (EP) of the experiment. Scale bar, 10 µm. **(b-e)** Quantitative analysis of total cell size **(b)**, cell body size **(c)**, capsule size **(d)**, and relative capsule size (**e**) in organs of H99-infected mice. Relative capsule size was calculated by dividing observed capsule size by whole cell size. The data shown are cumulative from more than 100 fungal cells representing four mice per group. Error bars indicate the 95% confidence interval of the median. Statistical analysis was performed with Tukey’s multiple comparisons test. ***, P < 0.001.

During *C. neoformans* infection, both cell body and capsule sizes undergo significant changes in pulmonary and extrapulmonary organs [32,35,36]. Titan cells are induced during the early stage of pulmonary infection and can persist for a long period, however, the proportion of titan cells decreases as the infection processes [13,14,28]. The abundance of small cells (total diameter ≤10 μm) increases at 10 days post-inoculation (dpi), coinciding with the dissemination of *C. neoformans* to the brain [40], suggesting a critical role of small cells in fungal spread and colonization [35]. We intranasally inoculated female C57BL/6 mice with the wild type strain H99 and measured the cell body and capsule sizes of fungal cells recovered the lungs, spleens, and brains of symptomatic mice euthanized at the endpoint (EP) (Fig 1). Measurements were also taken from lung samples at 3 dpi. Fungal populations in the spleen and brain were relatively uniform and small, whereas greater heterogeneity was observed in lung samples at EP (Fig 1a). Both the median total diameter (14.64 μm at EP vs 22.99 μm at 3 dpi) and cell body size (5.74 μm at EP vs 8.96 μm at 3 dpi) of fungal cells recovered from lungs were significantly smaller at EP than at 3 dpi (Fig 1b-1c). At EP, cell body size, capsule size, and the total size were smaller in the brain and spleen compared to fungal cells in the lungs. The median fungal cell body diameters were similar between brain and spleen samples (3.58 μm in the brain vs 3.71 μm in the spleen), but relative capsule sizes differed (Fig 1b-1e). These findings suggest that small cells not only facilitate extrapulmonary dissemination but also confer a growth advantage in the late stages of cryptococcosis.

### Host related conditions trigger the formation small-sized cell

Cell morphology changes in response to host conditions during *C. neoformans* infection. Host factors, such as nutrient starvation, pH, CO_2_, serum, and components of host microbiome, play important roles in titan cells formation. The establishment of *in vitro* culture conditions has helped elucidate the underlying mechanism of cells size regulation in *C. neoformans*. To identify factors involved in small cells production, we tried various host environmental conditions during *C. neoformans* infection. Nutrient limitation and high CO_2_ levels are the common conditions in the host, especially during the late stages of cryptococcosis, therefore, we examined cell morphotypes on media cultured at 37°C in 5% CO_2_ or under normal atmospheric conditions. Rich media were diluted to 100%, 50%, 25%, 10%, 5%, 2%, 1%, 0.5%, and 0% of their original concentration to generate different nutrient levels. The media used included mammalian cell culture media, such as Dulbecco’s modified Eagle medium (DMEM) and Roswell Park Memorial Institute 1640 (RPMI1640) medium, as well as the standard yeast culture medium yeast extract peptone dextrose (YPD).

**Fig 2:**
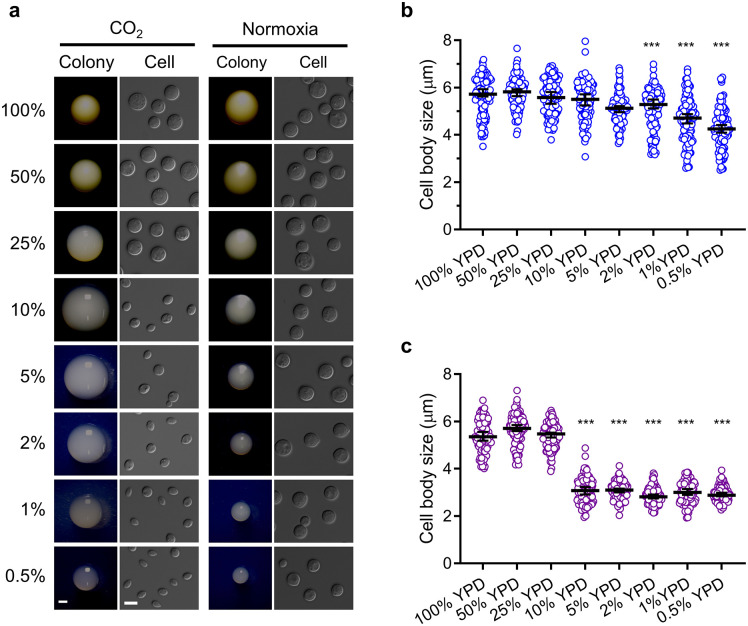
Colony and cellular morphologies on different concentration of YPD plates at 37°C for 5 days in 5% CO_2_ or under normoxia. **(a)** Representative images of colonies and cells of *C. neoformans* grown on 100%, 50%, 25%, 10%, 5%, 2%, 1%, and 0.5% YPD. Scale bar for colony, 1mm, scale bar for cell, 5 µm. **(b-c)** Quantitative measurement of cell body size from cells grown on different concentration of YPD plates in normal air (**b**) or in 5% CO_2_ **(c)**. Data are compiled from 100 cells per group. Error bars indicate 95% confidence interval of the median. Statistical analysis was performed with Tukey’s multiple comparisons test. ***, P < 0.001.

The wild type strain H99 was plated onto agar media with different concentrations of DMEM, RPMI1640, and YPD and cultured for 5 days. We observed that colony and cell sizes decreased with reducing nutrient concentrations and 0% rich media exhibit no visible colonies. CO_2_ played an important role in the production of small cells under low-nutrient conditions across all three media types (S1, S2 Figs and Fig 2). Thus, YPD was selected for further studies on small cell formation. Cells grown in normal air showed decreased colony sizes with reduced YPD concentration (Fig 2a), along with a slight reduction in median cell diameter (Fig 2b). In contrast, cells grown on 10% YPD and 5% YPD showed larger colony sizes compared to other YPD concentrations in the presence of 5% CO_2_ (Fig 2a). The median cell sizes in 5% CO_2_ were 5.35 µm, 5.71 µm, and 5.50 µm on 100%, 50%, and 25% YPD, respectively. The cell sizes were comparable to those grown under normal air. However, on media containing 10% to 0.5% YPD under CO_2_, median cell diameters were less than 3.08 µm, significantly smaller than those grown on nutrient rich conditions (100%-25% YPD) in the presence or absence of CO_2_ or nutrient limit conditions (10%-0.5% YPD) in air (Fig 2c). These findings indicate that both nutrient limitation and elevated CO_2_ levels promote small cell formation in *C. neoformans*. The majority of cells (approximately 95%) grown on 10% YPD agar in 5% CO_2_ for 5 days were small-sized. Therefore, this condition was used to induce the production of small cells (<4µm), which we designated as “ovoid cells” in this study.

*C. neoformans* infection acidifies the microenvironment. pH decreased to as low as 6.3 in infected lungs and 5.5 in infected brains [35,41]. pH influences the formation of both seed cells and titan cells [20,35]. To test whether pH regulates ovoid cell formation under *in vitro* condition, we cultured cells on 10% and 100% YPD media at different pH levels, which were buffered with KH_2_PO_4_. We observed reduced small cell formation at pH 4.0 and cell sizes were small at pH 5.0 to 8.0 on 10% YPD under CO_2_. Whereas cells grown on 10% YPD in air or on 100% YPD in CO_2_ showed typical size (S3 Fig). These findings suggest that ovoid cell formation is not significantly affected by pH, which is different from the development of seed cells.

### Analysis of gene expression profiles in ovoid cells

**Fig 3:**
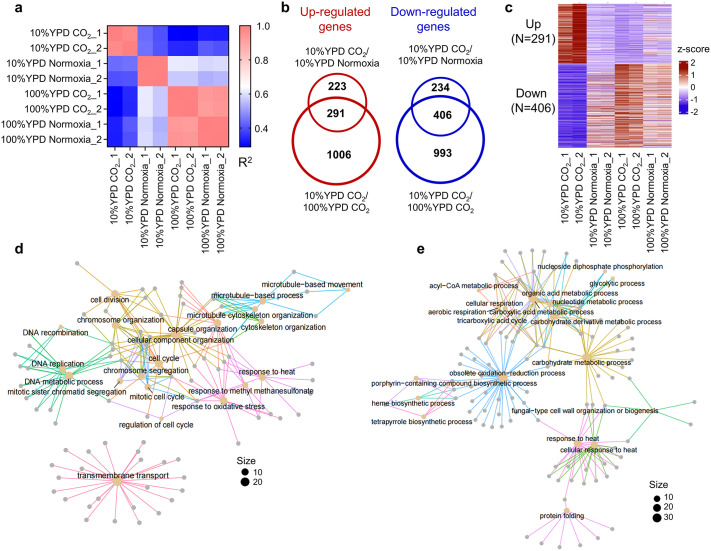
RNA-seq analysis of cells grown under ovoid cells inducing condition. **(a)** Correlation coefficient of gene expression profiles from cells grown on 100% YPD or 10% YPD in the presence or absence of 5% CO_2_ at 37 °C for 5 days. Clustering analysis was performed using the R package plots v3.1.3 (https://cran.r-project.org/web/packages/gplots/). Color intensity from blue to red (levels 0.3-1) indicates increasing consistency. R^2^: Square of Pearson correlation coefficient. **(b)** Venn diagram showing the numbers of differentially expressed genes in two comparison groups (10% YPD CO_2_ vs 10% YPD air and 10% YPD CO_2_ vs 100% YPD CO_2_) and the overlap between these differentially-expressed genes among two treatments. A 2-fold change cutoff (log2(x) = 1) was used to define differential expression. **(c)** Heatmap of overlapping differentially expressed genes between the two comparison groups (10% YPD CO_2_ vs 10% YPD normoxia and 10% YPD CO_2_ vs 100% YPD CO_2_) following k-means clustering based on expression values. Numbers in brackets indicate gene counts per cluster and the color scale represents row-wise Z-scores. **(d)** Top twenty most significantly enriched gene ontology (GO) terms for biological processes upregulated in ovoid cells. **(e)** Top twenty most significantly enriched gene ontology terms for biological processes downregulated in ovoid cells.

To understand the global gene expression landscape of ovoid cells, we performed RNA-Seq to compare transcriptomic profiles of H99 cells grown on 100% YPD or 10% YPD in the presence or absence of 5% CO_2_. Correlation coefficient analyses revealed that cells grown on 100% YPD in 5% CO_2_ and in air had largely overlapping global gene expression patterns. In contrast, cells grown on 10%YPD in CO_2_ showed differences in gene expression compared to cells grown on 10% YPD in air (Fig 3a). Ovoid cells grown on 10% YPD in CO_2_ exhibited more differentially expressed genes (DEGs) when compared to cells grown on 100% YPD in CO_2_ and cells grown on 10% YPD in air. 1297 genes were upregulated and 1399 genes were down-regulated in ovoid cells compared to cells grown on 100%YPD in CO_2_. When compared to cells grown on 10% YPD in air, 514 genes were up-regulated and 643 genes were downregulated in ovoid cells (Fig 3b). A total of 697 common DEGs were identified when comparing ovoid cells to cells grown on other two conditions. Among these shared genes, 291 genes were upregulated and 406 genes were downregulated in both comparison sets (10% YPD CO_2_ vs 10% YPD normoxia and 10% YPD CO_2_ vs 100% YPD CO_2_) (Fig 3c). Gene Ontology analysis of the common DEGs revealed that upregulated genes were involved in DNA replication, chromosome organization, cellular component organization, cell cycle regulation, transmembrane transport, and response to heat (Fig 3d and S1 Table), while downregulated genes were associated with carbohydrate metabolic process, oxidation-reduction process, and response to heat (Fig 3e and S1 Table).

Ovoid cells expressed high levels of cell cycle related genes, including genes involved in chromosome segregation (CNAG_07635_*NDC80*, CNAG_06408_*DCC1*, CNAG_01959, CNAG_04936, CNAG_00681), DNA replication and recombination (CNAG_07909_*DMC1*), cell division (CNAG_00680, CNAG_01983_*RCV1*, CNAG_04026), and regulation of the cell cycle (CNAG_01882, CNAG_06697_*MPS1*, CNAG_03184_*BUB1*) (S1 Table). Consistent with cell cycle regulation, ovoid cells also showed upregulated genes related to cytoskeleton organization (S1 Table). Genes encoding transmembrane transporters for amino acid and nicotinic acid were induced in ovoid cells. Two phosphate transporters CNAG_02777 (*PHO84*) and CNAG_05459 (*PHO840*) are upregulated, while two mitochondrial phosphate transporters (CNAG_06377 and CNAG_03824) were downregulated in ovoid cells. The downregulation of both *PHO84* and *PHO840* in seed cells and their upregulation in ovoid cells indicate that these two cell types are distinct [35].

Enzymes involved in carbohydrate metabolism were repressed in ovoid cells (Fig 3e and S1 Table). These included glycolysis related genes, such as hexokinase (CNAG_03769_*HXK2*), fructose-bisphosphate aldolase (CNAG_06770_*FBA1*), and phosphopyruvate hydratase (CNAG_06868), as well as genes affecting the tricarboxylic acid cycle (TCA cycle), such as malate dehydrogenase (CNAG_03225_*MDH1*), citrate synthase (CNAG_00061_*CIT1*), ketoglutarate dehydrogenase (CNAG_03596_*KGD1*), and succinate dehydrogenases (CNAG_04189_*SDH1*, CNAG_03226_*SDH2*, CNAG_06732_*SDH4*). Additionally, 6-phosphogluconolactonase (CNAG_02133_*SOL3*) and 6-phosphogluconate dehydrogenase (CNAG_04099 and CNAG_07561), which belong to the pentose phosphate pathway, were down-regulated in ovoid cells. Genes encoding enzymes involved in cell wall organization were also reduced, including three chitin deacetylases (CNAG_05799_*CDA1*, CNAG_01230_*CDA2*, CNAG_01239_*CDA3*) and four out of six ricin B-containing proteins (CNAG_00587, CNAG_00588, CNAG_02526, and CNAG_04891) in *C. neoformans*.

The gene CNAG_06312 (encoding the ovoid cell specific protein Osp1) was highly expressed in ovoid cells grown on 10% YPD in CO_2_ with an FPKM value exceeding 500,000. In contrast, FPKM<10 in cells grown on 100% YPD in CO_2_ (S4a Fig). To visualize Osp1 expression, we generated a strain expressing Osp1:mCherry fusion protein under the control of its native promoter. This strain was cultured for 5 days on 10% YPD or 100% YPD in CO_2_, or on 10% YPD under normoxia. A strong fluorescent signal was observed in ovoid cells, while no signal was detected under the other two conditions. Additionally, normal-sized cells produced on 10% YPD in CO_2_ also showed no signal (S4b Fig). Using *OSP1* expression as a marker, we found that ovoid cells constitute approximately 95% of the cell population when the wild type strain H99 was cultured on 10% YPD agar in 5% CO_2_ at 37 °C for 5 days (S4c Fig). We also examined fluorescence in the mCherry tagged strain cultured in liquid media. Fluorescent signals were detected in cells grown in liquid 10% YPD under 5% CO_2_, as well as in a few small cells grown in 10% YPD under normoxia (S4d Fig). Furthermore, the mCherry tagged strain was also used to assess expression in seed cells and titanides. No fluorescence was observed in either cell types, indicating that *OSP1* is specifically expressed in ovoid cells and that this cell type is distinct from both seed cells and titanides.

### Glucose levels influence ovoid cells formation

The downregulation of carbohydrate metabolism related genes in ovoid cells prompted us to investigate the role of carbon sources in ovoid cells formation (Fig 3e and S1 Table). We hypothesized that glucose (D) is critical for ovoid cell induction, although other components of YPD, including yeast extract (Y) and peptone (P), can also serve as carbon sources for *C. neoformans*. To determine the base concentration of YP, we assessed cell morphotypes on plates containing 100%, 50%, 25%, 10%, 5%, 2%, and 1%, and 0.5% of the original YP levels in YPD medium (S5 Fig).

**Fig 4:**
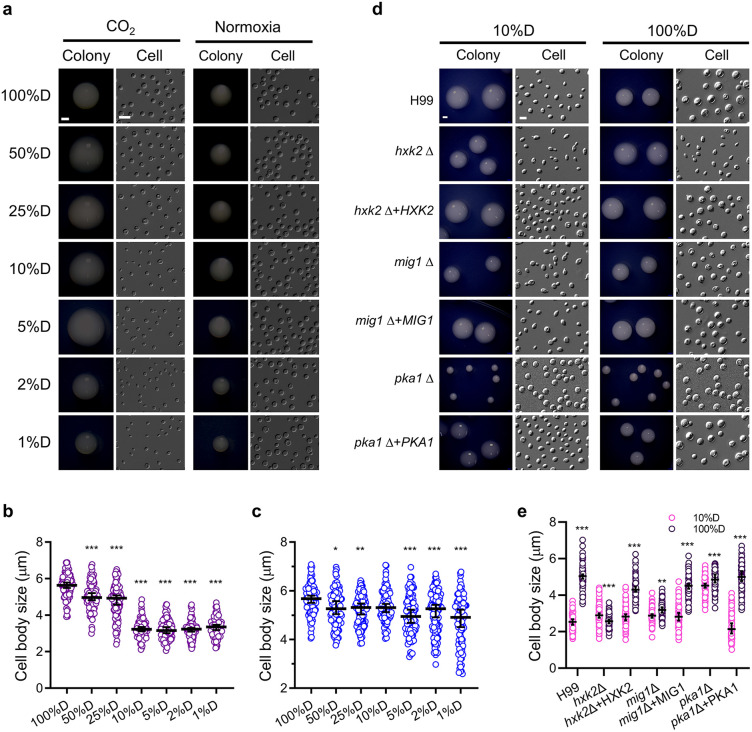
Roles of the glucose sensing and cAMP/PKA pathways in ovoid cell production. **(a)** Representative colony and cell images of *C. neoformans* grown on 10% YP supplemented with different glucose concentrations for 5 days in 5% CO_2_ or in air at 37°C. D, Dextrose. Scale bar for colony, 1mm, scale bar for colony, 5 µm. **(b-c)** Quantitative measurement of cell body size from cells grown 10% YP with different concentrations of D in 5% CO_2_ (**b**) or under normoxia **(c)**. Data are compiled from 100 cells per group. Error bars indicate 95% confidence interval of the median. *, P < 0.05, **, P < 0.01 ***, P < 0.001. **(d)** Colony and cellular morphologies of *HXK2*, *MIG1*, or *PKA1* deletion strains grown in CO_2_ for 5 days. Scale bar for colony, 1mm, scale bar for colony, 5 µm. **(e)** Quantitative measurement of cell body size from *HXK2*, *MIG1*, or *PKA1* deletion mutants and gene complemented strains grown on 10% YP with 10% or 100% D in 5% CO_2_. Data are compiled from 100 cells per group. Error bars indicate the 95% confidence interval of the median. **, P < 0.01, ***, P < 0.001.

Ovoid cell populations were present at 100%, 50%, and 25% YP, indicating that high glucose levels inhibit ovoid cells production. Cells grown on agar containing 10% or less YP presented as ovoid cells (S5 Fig). Using 10% YP as the base conditioned medium, we examined how glucose regulates ovoid cell formation and found a negative correlation between ovoid cell production and glucose concentration in the presence of CO_2_ (Fig 4a). The 10% YP + 10% D medium promoted the formation of ovoid morphotypes under 5% CO_2_. In contrast, cells grown on 10% YP + 10% D under normoxia or on 10% YP + 100% D with or without CO_2_ showed typical cell sizes (Fig 4b-4c). Additionally, varying the pH from 5.0 to 8.0 in 10% YP based media did not affect ovoid cell formation in 5% CO_2_ (S6 Fig).

The encoding gene of hexokinase (*HXK2*, CNAG_03769), which were identified in *Saccharomyces cerevisiae* as multifunctional protein that functions as glycolytic enzyme catalyzing glucose phosphorylation to glucose-6-phosphate and as a regulator of Mig1 controlling glucose repression [42–44], was downregulated in ovoid cells (S1 Table). To examine the role of *HXK2* in ovoid cell formation, we generated a *HXK2* deletion mutant and cultured *hxk2*∆ cells on 10% YP + 10% D or 10% YP + 100% D in the presence or absence of CO_2._ Ovoid cells of *hxk2∆* were observed on 10% YP + 100% D medium under 5% CO_2_ (Fig 4d), indicating that *HXK2* negatively regulates ovoid cells production. Although no differential expression of *MIG1* was observed between ovoid cells and typical cells, *mig1*∆ cells showed a shift toward ovoid morphology on 10% YP + 100% D (Fig 4d), confirming that glucose repression signaling promotes ovoid cell formation. The cAMP/PKA signaling pathway is involved in glucose sensing and glycolysis in *C. neoformans* [45]. Disruption of *PKA1* resulted in smaller colony sizes compared to H99 under ovoid cell induction conditions and no ovoid cells were observed in the *pka1*∆ mutant (Figs 4d-4e and S7), suggesting that the cAMP/PKA pathway plays an important role in regulating small cell size.

Both seed cells and titanides exhibit a cell shape similar to that of ovoid cells. To investigate whether genes involved in regulating seed cells or titan cells also play a role in ovoid cell formation, we examined the ovoid cell production ability of the *rim101*Δ, *crk1*Δ, *fbp1*Δ, and *pho4*Δ mutants. Rim101 and Crk1 positively regulate titan cell formation [27,28], whereas Fbp1 acts as a negative regulator of titan cell development [28]. Deletion of *PHO4* reduces titan cell formation and promotes seed cell production in a pH dependent manner [35]. We found that the *fbp1*Δ mutant exhibited normal cell size when grown on 10% YPD in CO_2_, comparable to cells grown on 100% YPD in CO_2_. The *rim101*Δ, *crk1*Δ, and *pho4*Δ mutants retained the ability to produce ovoid cells similar to H99 (S8 Fig). These results suggest that genes regulating titan cell formation may also be involved in the development of ovoid cells.

### Features of ovoid cells induced under host related conditions

**Fig 5:**
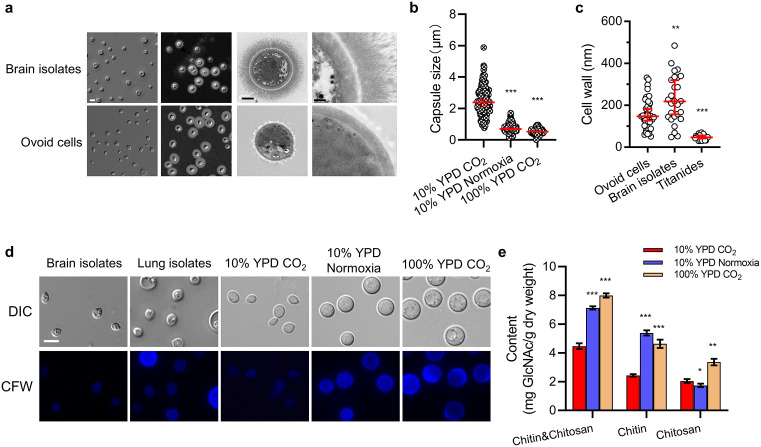
Characteristics of ovoid cells. **(a)** Representative cellular and TEM images of *C. neoformans* cells isolated from infected brains and ovoid cells. Scale bar, 10 µm, 1 µm and 200 nm. **(b)** Quantitative measurement of capsule size of cells grown under the indicated conditions. The data shown are cumulative from 100 cells. Error bars represent the 95% confidence interval of the median. Statistical analysis was performed with Tukey’s multiple comparisons test. ***, P < 0.001. **(c)** Quantitative measurement of cell wall thickness of ovoid cells, brain isolates, and titanides. The data shown are cumulative from over 20 cells. Error bars indicate the 95% confidence interval of the median. Statistical analysis was performed with Tukey’s multiple comparisons test. **, P < 0.01, ***, P < 0.001. **(d)** CFW staining of cells isolated from the brain and lung of infected mice, as well as cells grown on 100% YPD or 10% YPD with or without CO_2_. DIC, differential interference contrast, CFW, Calcoﬂuor white. Scale bars, 5 µm. **(e)** Quantitative measurement of chitin/chitosan, chitin, and chitosan levels in cells grown on 100% YPD or 10% YPD with or without CO_2_. Data are combined from three independent experiments. Error bars indicate the standard error of the mean. Statistical analysis was done between normal-sized cells and ovoid cells by a two-tailed t test. *, P < 0.05, **, P < 0.01, ***, P < 0.001.

Cells from organs of infected mice at EP and ovoid cells showed similar cell morphological characteristics that populations contain cells of ovoid shape and small size. Both cell collections showed high heterogeneity in capsule size (Fig 5a-5b). Transmission electron microscope (TEM) images revealed the cell wall thickness of ovoid cells, fungal cells isolated from infected brains, and titanides (Fig 5a and 5c). Most brain isolates possessed dense and structured capsules, whereas the capsule of ovoid cells was often damaged during TEM processing (Fig 5a). The cell wall of ovoid cells was thicker than that of titanides and some ovoid cells exhibited similar cell wall thickness compared to that of brain isolates (Fig 5c). Calcofluor white (CFW) staining, which was used to assess chitin content in the cell wall, showed that small cells grown on 10% YPD in CO_2_ or isolated from brains or lungs exhibited reduced fluorescence signals compared to cells cultured on 100% YPD or on 10% YPD in air (Fig 5d). Quantitative analysis chitin and chitosan content in cells cultured under different conditions indicated that both chitin and chitosan levels were lower in ovoid cells compared to cells grown on 100% YPD in CO_2_. The nucleolar protein Nop1 fused to a fluorescent protein was used to visualize nuclei [46]. The signal area and the diameter ratio of nuclear to cell body were smaller in ovoid cells compared to normal sized cells (S9a Fig). DAPI staining confirmed that small cells had smaller nuclei than normal sized cells (S9b Fig). CFW staining and nuclear visualization of seed cells and titanides exhibited no difference compared to ovoid cells. These observations suggest that ovoid cells share similarities, but also possess distinct features, with previously reported small cells such as seed cells and titanides.

### Ovoid cells exhibit growth advantages under nutrient limitation condition in the presence of CO_2_

**Fig 6:**
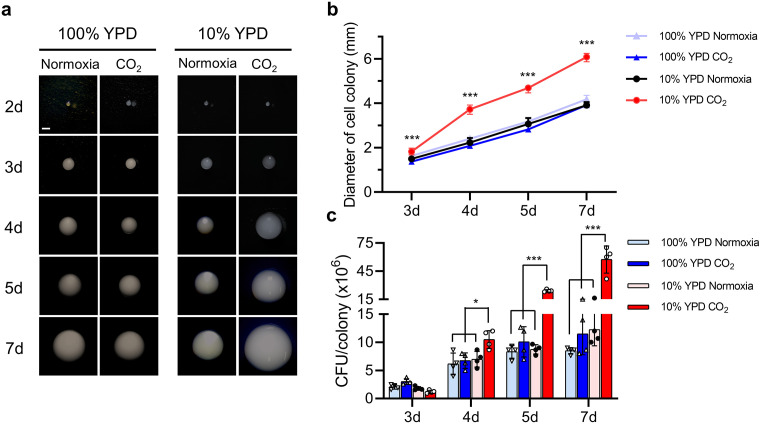
Growth rate of ovoid cells under *in vitro* culture conditions. **(a)** Representative colony images to show the growth of cells on 100% YPD or 10% YPD with or without CO_2_ at indicated time points. Scale bar, 1mm. **(b)** Changes in colony diameter after growing H99 cells on 100% YPD or 10% YPD for 3 days to 7days. Error bars represent standard deviations of fifteen colonies. Statistical analysis was done between normal-sized cells and ovoid cells by a two-tailed t test. ***, P < 0.001. **(c)** Quantification of CFUs per colony of H99 grown under the indicated conditions. CFUs were counted from four colonies per group. Error bars indicate standard deviations of three repeats. Statistical analysis was performed with Tukey’s multiple comparisons test. ***, P < 0.001.

The observation that colony sizes on 10% YPD and 5% YPD plates are larger than those on other YPD concentrations led us to investigate the growth advantages of ovoid cells. *C. neoformans* cells were plated on 100% YPD or 10% YPD and cultured in 5% CO_2_ or in ambient air for 7 days. Colonies were imaged daily and colony forming units (CFU) were counted each day. Cells grown on 100% YPD with or without CO_2_ and on 10% YPD in air showed similar growth rates. In contrast, cells grown on 10% YPD in CO_2_ showed larger colony diameters than those under other conditions after 3–7 days of culture (Figs 6a-6b and S10). CFU counts revealed that colonies grown on 10% YPD in CO_2_ had 1.50-, 2.76-, and 4.69-fold higher CFUs than those grown on 10% YPD in air after for 4, 5, and 6 days, respectively (Fig 6c). These data suggest that ovoid cells have a growth advantage under host related conditions, including low nutrient and high CO_2_ levels.

### Ovoid cells are tolerant to fluconazole

**Fig 7:**
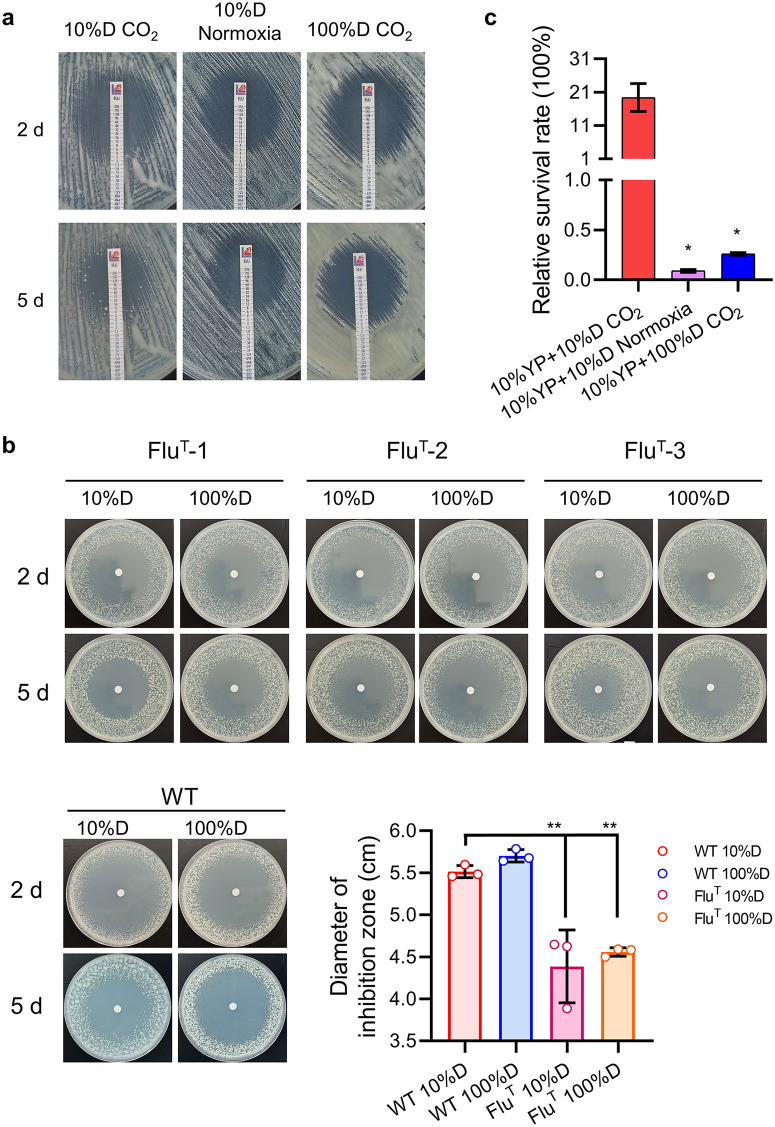
Ovoid cells show tolerance to fluconazole. **(a)** E-test strips were used to test the MIC to fluconazole. Cells were streaked onto 10% YP agar supplemented 10% or 100% glucose and E-test strips with fluconazole were placed on the plates. Plates were incubated for 2 or 5 days with or without CO_2_. Images shown are representative of three independent experiments. **(b)** Fluconazole susceptibility of colonies grown within the inhibition zone. Five microliters of 50 mg/mL fluconazole were dropped onto a paper disc in the middle of plates. The diameters of inhibition zone were measured after 2 days of incubation. Flu^T^, Fluconazole tolerance strain. Error bars indicate the standard error of the mean. Statistical analysis was done by a two-tailed t test. **, P < 0.01. **(c)** Tolerance rate of cells after treatment with a high concentration of fluconazole. Cells collected from indicated conditions were resuspended into liquid medium as indicated and treated with 80 μg/mL fluconazole for 6 days. The rate was calculated as the ratio of CFUs between treated and untreated samples. Error bars indicate the standard error of the mean. Statistical analysis was done by a two-tailed t test. *, P < 0.05.

Fluconazole, a triazole antifungal drug, is commonly used to treat cryptococcosis [47]. *C. neoformans* generally has a low MIC to fluconazole and strains with high minimum inhibitory concentration (MIC) are associated with clinical treatment failure [48]. We tested the MIC of fluconazole for ovoid and typical cells of H99 using E-test strips. H99 cells were plated onto 10% YP + 10% D or 100% D and cultured in CO_2_ or under normoxia. Cells grown under different conditions showed the same MIC after 2 days of culture (Fig 7a). However, after 5 days of culture, cells grown on ovoid cell inducing condition exhibited more colonies within the inhibition zone compared to cells grown on 10% YP + 10% D in air or on 10% YP + 100% D in 5% CO_2_ (Fig 7a). We collected several colonies from the zone of inhibition and assessed their susceptibility to fluconazole using the disc diffusion method. Smaller inhibition zones were observed for these fluconazole tolerant strains, even when cells were grown on 10% YP + 100% D in 5% CO_2_. Additionally, an increased number of colonies within the inhibition zone was observed when tolerant cells were cultured under ovoid cell inducing condition (Fig 7b). Given that fluconazole has time-dependent and prolonged antifungal effects [49], we tested the tolerance rate of ovoid and typical cells under high concentrations of fluconazole treatment over long time period. Ovoid cells exhibited a significantly higher tolerance rate compared to typical cells (Fig 7c). These data suggest that ovoid cells are tolerant to fluconazole and may influence the efficacy of clinical fluconazole treatment.

### Ovoid cells exhibit attenuated fungal virulence

To assess the virulence of *C. neoformans* ovoid cells, we first performed a murine inhalation model of systemic cryptococcosis using C57BL/6 female mice (S11 Fig). H99 cells were collected from 100% YPD or 10% YPD plates after 5 days of growth in 5% CO_2_ or in air. Cells from H99 overnight culture in liquid YPD at 30°C served as the control. Mice were infected with 10^5^ cells of each strain and surveilled for survival. Mice infected with either initial ovoid or typical cells showed similar survival rates that all died within 28 days (S11a Fig). The fungal burden in the brain, lung, and spleen of infected mice showed no significant difference at the endpoint (23 dpi) (S11b Fig). We also examined the fungal burden of lung tissues after 1-, 3-, and 7-days post-infection and found no difference among the different initial cell types (S11c Fig).

To investigated the biological relevance of ovoid cells and determine whether mouse strain influences ovoid cell production during infection, we intranasally inoculated both BALB/c and C57BL/6 female mice with ovoid cells and typical cells of the Osp1:mCherry strain. Initial ovoid cells exhibiting strong fluorescence signals were collected from cultures grown on 10% YPD in CO_2_, while typical cells produced on 100% YPD in CO_2_ showed no mCherry signal. Both ovoid cells and typical cells appeared enlarged and no fluorescence was detected in lung isolates at 3 dpi (S11d Fig), suggesting that ovoid cells development is blocked during the early stage of *Cryptococcus* infection in the murine inhalation model. Large cells were observed in infected lungs at 3 dpi, whereas small cells were predominated in lungs at the endpoint (S11e Fig). mCherry signals were detected in cells isolated from the brains, lungs, and spleens of mice infected with the tagged strain at the endpoint (S11d Fig), indicating that ovoid cells are produced during the late stage of cryptococcosis and that their production is independent of organs and mouse strain.

**Fig 8:**
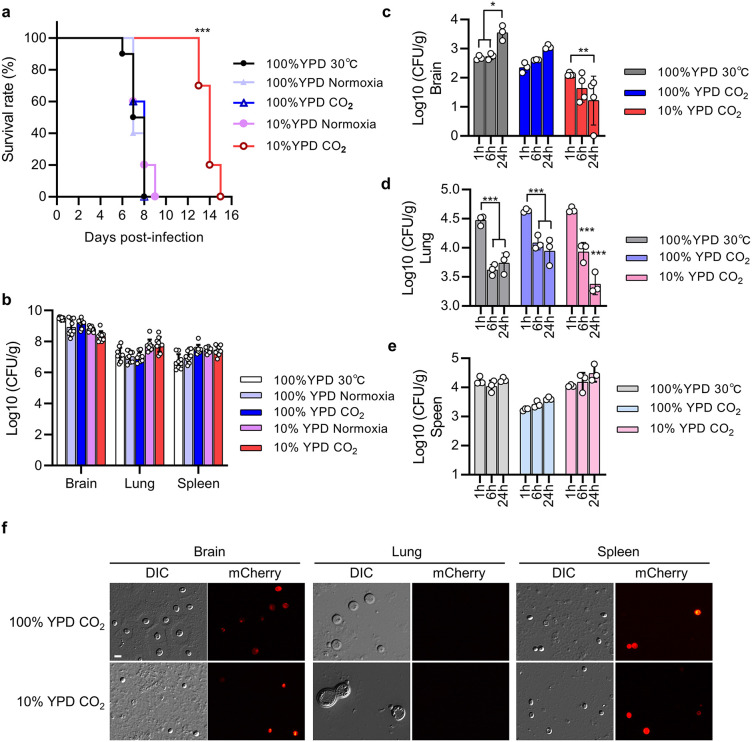
Virulence of ovoid cells in a tail vain injection model. **(a)** Survival curves of mice injected via tail vein injection with cells grown under indicated conditions. Statistical analysis was performed based on Log-rank (Mantel-Cox) test. ***P < 0.001. **(b)** Fungal burden in organs of infected mice at the endpoint. Data are pooled from 10 mice per group. Error bars indicate the 95% confidence interval of the median. **(c-e)** Fungal burden in the brains **(c)**, lungs **(d)**, and spleens (e) of mice at 1-, 6-, and 24-hours post-infection. The data shown are compiled from 3-4 mice per group. Error bars indicate the standard error of the mean. Statistical analysis was done by a two-tailed t test. *, P < 0.05, **, P < 0.01, ***, P < 0.001. **(f)** Representative images to show ovoid cells in brain, lung and spleen of infected mice. DIC, differential interference contrast. Scale bar, 10 µm.

*C. neoformans* undergoes dramatic changes in cell size in the lungs during intranasal infection [14]. The instability of ovoid cells induced by the wild type strain in the murine inhalation model may affect the accuracy of assessing fungal virulence of ovoid cells. We therefore established a systemic infection model via tail vein injection in BALB/c female mice (Fig 8). Mice infected with H99 typical cells grown on 100% YPD or 10% YPD under normoxia exhibited survival rates similar to those of cells collected from liquid YPD cultures at 30°C (Fig 8a). In contrast, mice infected with ovoid cells survived significantly longer, indicating attenuated virulence of the ovoid cells (Fig 8a). No difference in fungal burden were observed in the brain, lung, or spleen of tail vein injected mice euthanized due to endpoint symptoms, suggesting that the differential virulence between ovoid cells and typical cells was established at the early stage during infection (Fig 8b). We then examined cell colonization in the brain, lung, and spleen at 1, 6, and 24 hours after tail vein injection (Fig 8c-8e). At 1 hour post infection, ovoid cells and typical cells from the wild type strain showed similar colonization levels in each organ. However, the two cell types exhibited fungal burden differences after 6 and 24 hours of infection. The fungal burden of typical cells increased in the brains and spleens over time, but decreased in the lungs after 6 hours and remained stable at 24 hours. Although the fungal burden of ovoid cells increased in the spleen, they showed significantly reduced colonization in the brains and lungs over time. Thus, ovoid cells represent a virulence attenuated cell type that is less capable of colonizing the brain and lungs. We also assessed ovoid cell production in the tail vein injection model using Osp1:mCherry strain and found that typical cells were able to produce ovoid cells in the brain and spleen at 3 dpi. No fluorescence signals were detected in the lungs of mice infected with either typical cells or ovoid cells, suggesting that the host lung environment affects ovoid cells production.

### Ovoid cell formation is unique to *C. neoformans*

**Fig 9:**
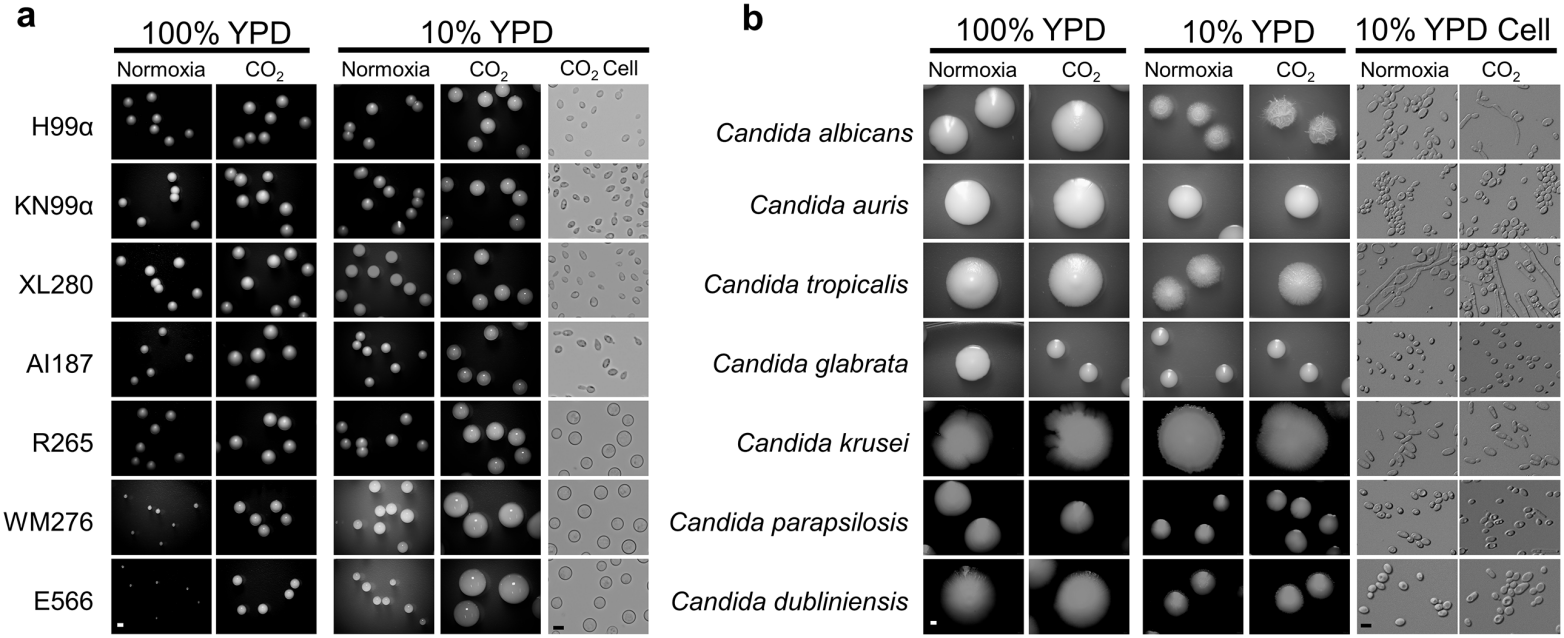
Colony and cellular morphologies of *Cryptococcus* and *Candida* species under *in vitro* ovoid cell induction condition. **(a)** Representative colony and cell images of *C. neoformans* and *C. gattii* species. *C. neoformans*: H99α and KN99α (Serotype **A)**, XL280 (serotype **D)**, AI187 (diploid). *C. gattii*: R265, WM276, and E566 (serotype **B)**. Scale bar for colony, 1mm, scale bar for cell, 5 µm. **(b)** Representative colony and cell images of *Candida* species. Scale bar for colony, 1mm, scale bar for cell, 5 µm.

Ovoid cells are associated with fungal virulence and antifungal drug resistance in *C. neoformans*. To determine whether other species can produce ovoid cells under the low nutrient and high CO_2_ conditions, we utilized *in vitro* ovoid cell induction condition to test various species, including *C. neoformans* and *C. gattii* of different serotypes, as well as human fungal pathogens within the *Candida* species (Fig 9). Cells were grown on 100% YPD and 10% YPD in 5% CO_2_ or in air for 5 days. We observed ovoid cells in serotype A, D, and even diploid strains of *C. neoformans* (Fig 9a). None of the tested strains, representing *C. gattii*, *C. albicans*, *C. auris*, *C. tropicalis*, *Candida galbrata*, *Candida krusei*, *Candida parapsilosis*, and *C. dubliniensis*, produced ovoid cells under the *in vitro* ovoid cell induction condition (Fig 9a-9b). These findings suggest that ovoid cell type formation is a rare phenomenon specific to *C. neoformans*.

### The ability to form ovoid cells in clinical *C. neoformans* isolates

**Fig 10:**
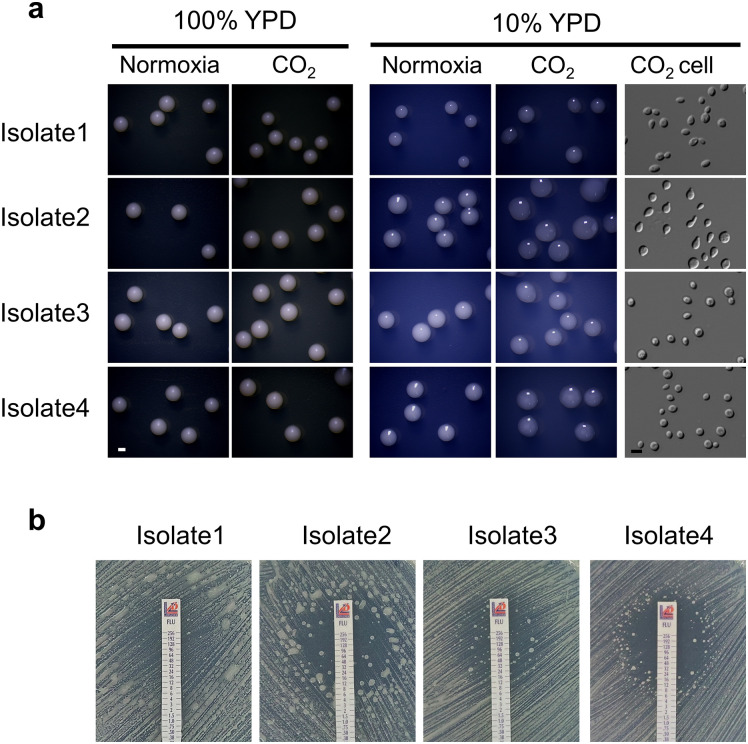
Clinical isolates of *C. neoformans* can produce ovoid cells. **(a)** Colony and cell images of four *C. neoformans* clinical isolates grown on 100% YPD or 10% YPD in the presence or absence of CO_2_. Scale bar for colony, 1mm, scale bar for cell, 5 µm. **(b)** E-test strips were used to test the fluconazole MIC of four clinical isolates cultured under ovoid cell induction condition for 5 days.

Infection by *C. neoformans* occurs via inhalation of environmental cells. Morphological changes of *C. neoformans* are important for the maintenance and progression of cryptococcosis, as well as the efficacy of antifungal treatment. We tested the capacity for ovoid cells formation in clinical isolates of *C. neoformans* collected from West China Hospital, Sichuan University. Four out of fifteen strains were able to produce ovoid cells under *in vitro* ovoid cell induction condition (Fig 10a). Similar to the H99 strain, all four clinical isolates formed colonies within the halo of inhibition under ovoid cell induction conditions, whereas the other 11 strains which did not form ovoid cell showed no colonies in the inhibition zone (Fig 10b). These observations indicate that the ability of ovoid cells formation of *C. neoformans* clinical isolates suggests their tolerance of fluconazole.

## Discussion

Cellular heterogeneity of *C. neoformans* population contributes to its pathogenicity and antifungal drug resistance. The formation of large titan cells has been extensively studied and recognized as a novel virulence factor in *Cryptococcus* species [13,14]. Several types of small cells have also been identified under *in vivo* or *in vitro* conditions. Here we present a distinct small cell type, termed ovoid cell, which can be induced in *C. neoformans* under both *in vivo* and *in vitro* conditions such as low nutrient and high CO_2_ levels. Ovoid cells exhibit different features compared to other cell types of *C. neoformans*, including cell wall thickness, proliferation rate, and gene expression profiles. Osp1 was identified as an ovoid cell specific protein, which was not detected in either seed cells or titanides. We found that the glucose repression signaling pathway and the cAMP/PKA pathway are critical for ovoid cell formation in *C. neoformans*. Ovoid cell showed attenuated virulence during systematic intravenous infection and increased tolerance to fluconazole treatment.

Ovoid cells represent a morphotype distinct from other small cells, particularly in terms of cell shape. They have a larger cell body and a thinner cell wall compared to micro-cell. Ovoid cells differ from titanides in both cell wall thickness and *OSP1* expression levels indicating that ovoid cells and titanides are distinct cell populations. Although we observed differential expression of phosphate transporter genes between ovoid and typical cells, the production of ovoid cell was not influenced by variations in pH or KH_2_PO_4_ levels. Moreover, *OSP1* was not induced in seed cells, suggesting that ovoid cells are also distinct from this cell type. The difference in ovoid cell formation between the inhalation and tail vein injection models suggests that ovoid cells and seed cells exhibit different order during infection. Irregular cells, which have been observed in patients treated with antifungals, are thought to reflect a balance between fungal virulence and adaptation under antifungal therapy. These irregular cells are hypothesized to be defective [37]. The relationship between ovoid cells and irregular cells in *C. neoformans* requires further investigation.

Checkpoint control is well known mechanism for cell size regulation in proliferating cells, where growth and division are inherently coupled to determine cell size [50]. Cells divide only upon reaching a critical size, which is modulated by extracellular nutrient conditions. We found that poor nutrient lead to reduced cell size in *C.neoformans*, which is consistent with reports in other species such as *S. cerevisiae* [50,51]. Nutrients modulate cell size and growth via the TOR pathway in budding yeast. Tor1 kinase assembles distinct multiprotein kinase complexes called TORC1 and TORC2, both involved in the nutrient-mediated cell size control [52]. Gene expression analysis revealed no significant differences in *TOR1* expression between ovoid and typical cells. Moreover, cells grown under the same low nutrient conditions showed different sizes depending on CO_2_ exposure, indicating that mechanisms beyond the TOR pathway contribute to ovoid cells formation in *C. neoformans*. Mammalian host tissues contain higher CO_2_ levels than the external environment. CO_2_ tolerance is important for *C. neoformans* dissemination from the lungs to other organs and for fungal virulence [53–55]. The presence of 5% CO_2_ triggers titan cells and small-sized titanides production in *C. neoformans*, indicating that CO_2_ is involved in cell size regulation. In this study, we found that high concentrations of CO_2_ induce ovoid cell formation and cellular proliferation, indicating that CO_2_ is involved in cell size regulation in *C. neoformans*.

The ovoid cell type was observed only in *C. neoformans*, and not in *C. gattii* or *Candida* species, suggesting that ovoid cell formation may be a unique feature of *C. neoformans*. However, we only tested a limited number of strains, further investigation with additional fungal species and strains may yield more thorough picture. Previous studies examining titan cell production among basidiomycetous yeasts and *S. cerevisiae* found that titanization occurs primarily in the *C. neoformans* and *C. gattii* species complex [24,25]. Virulence factors, including melanin and capsule, are specifically produced in *Cryptococcus* species and contribute to pathogenicity. Thus, cellular heterogeneity as a virulence factor may be unique to *Cryptococcus* species. While *C. neoformans* yeast cells are typically round under standard laboratory conditions, *C. gattii* cells can exhibit round, oval, or ovoid shapes depending on the strain [56]. However, under the host related environment, *C. neoformans* produced ovoid cells, whereas tested *C. gattii* strains remained uniformly round. Consistent with this, a previous study to examine the correlation between morphological variability and outcome of *Cryptococcus* clinical isolates found that microcells occurred exclusively in *C. neoformans* [37]. These findings suggest that ovoid cell formation is likely an adaptive response of *C. neoformans* that facilitates cell proliferation during infection.

## Materials and methods

### Ethics statement

All animal studies were conducted in accordance with the guidelines for the Ethical Care of Laboratory Animals (No. 398, 2006) approved by the Ministry of Science and Technology of China and were approved by the Animal Ethics Committee of Southwest University. Mice were housed in groups of five in individually ventilated cages at 21 ± 1 °C, 30–70% relative humidity, 12 h/12 h dark/light cycle from 7:00 am-7:00 pm, with free access to food and water. Autoclavable mouse houses were provided as environmental enrichment.

### Statistics and reproducibility

Unless otherwise specified, all data presented in the figures are representative of at least three independent experiments with consistent results. Microscopy images shown are representative of three independent experiments. Specific statistical methods are described in the figure legends. The raw data in this study are listed in (S2 Table).

### Strains and media

The wild-type *Cryptococcus* strains and their derivatives used in this study are listed in (S3 Table). All primers used in this study are listed in (S4 Table). Yeast extract peptone dextrose (YPD) agar medium (1% yeast extract, 2% peptone, 2% dextrose, 2% agar) was used for the routine culture of *Cryptococcus* strains at 30 °C. Modified 10% YPD or 10% YP + 10% D agar media (0.1% yeast extract, 0.2% peptone, 0.2% dextrose, 2% agar) were used for inducing ovoid cells production. Dulbecco’s modified Eagle medium (DMEM) and Roswell Park Memorial Institute 1640 (RPMI1640) medium were prepared as described previously [57,58]. The media with different pH values were buffered using 4 g/L KH_2_PO_4_ and adjusted to the target pH with NaOH or HCl.

### Generation of deletion constructs

An overlap PCR recombination strategy was used to generate target-speciﬁc PCR products of the *SAT1* cassette flanked by 5’ and 3’ homologous arms of the deletion regions. The Cas9 expression cassette was amplified from the plasmid pXL1-Cas9. The gRNA target sequence was designed using the design tool in FungiDB (https://fungidb.org/fungidb/app/). The single guide RNA expression cassette (sgRNA) was constructed by fusing the U6 promoter and terminator through the gRNA sequence. Transient CRISPR-Cas9 coupled with electroporation system (TRACE) was used for *C. neoformans* transformation [59]. For positive selection of transformants, 500 µg/mL nourseothricin (clonNAT) was added to YPD solid medium. Correct transformants were verified by PCR assays.

### Electroporation of *C. neoformans*

Electroporation was performed as previously described [60]. Briefly, overnight cultures were transferred into 100 mL YPD at an initial OD_600_ around 0.2 and grown for 4–5 h at 30 °C until the OD_600_ reaches 0.6-0.8. All samples were kept cold in the following steps. Cells were collected, washed twice, resuspended in electroporation buffer (10 mM Tris-HCl pH 7.5, 1 mM MgCl2, 270 mM sucrose), and incubated with 1 mM DTT on ice for one hour. Cells were pelleted and resuspended in 250 μL electroporation buffer. 45 μL cells were mixed with 5 μL DNA and transferred to a precooled 2 mm gap cuvette. Electroporation was performed using an Eppendorf Multiporator with the following settings: V = 2 kv. Cells were resuspended in 1 mL YPD, transferred to 1.5 mL Eppendorf tubes, and incubated at 30 °C for 1.5 h before plating onto selective agar plates.

### DAPI staining

DAPI (4,6-diamidino-2-phenylindole) staining was performed as previously reported [57]. Briefly, cell cultures were fixed with 9.3% formaldehyde for 10 min. Fixed cells were washed twice with phosphate-buffered saline (PBS), permeabilized with an equal volume of PBS containing 1% Triton X-100 for 5 min, washed twice again with PBS, and resuspended in PBS. Equal volumes of cell suspension and DAPI solution (20 ng/mL DAPI) were mixed and incubated at 30°C for 45 min in the dark. Cells were washed twice with PBS and observed under a fluorescence microscope.

### Calcoﬂuor white staining

Cells were collected, washed, and resuspended in PBS (pH 7.4). Calcoﬂuor white (CFW, fluorescent brightener 28) was added to the cell suspension at a final concentration of 5 µg/mL and incubated for 10 min in the dark. Cells were washed twice with PBS and imaged using a fluorescence microscope.

### Chitin and chitosan measurement

*Cryptococcus* chitin and chitosan measurements were done as described previously [61]. Briefly, fungal cultures were collected divided, and dry weights were measured. One aliquot of pelleted cells was treated with sodium bicarbonate and acetic anhydride at room temperature for 20 min, followed by 5 min at 100 °C. Both cell aliquots were subsequently extracted with KOH at 80 °C for 90 min. Samples were collected and suspended in 0.2 ml of McIlvaine’s buffer (0.2 M Na_2_HPO_4_, 0.1 M citric acid, pH 6.0) containing 10 μg of chitinase from *Trichoderma viride* and incubated for 2 days at 37 °C. For colorimetric determination of N-acetylglucosamine (GlcNAc), the Morgan-Elson method was adapted for microplate readers. One hundred microliters of each sample were transferred to 96-well low-evaporation microliter plates, and absorbance at 585 nm was recorded. Standard curves were prepared from stocks of 0.2 to 2.0 mM GlcNAc. The data shown are cumulated from three independent experiments. Statistical analysis was done by a two-tailed t test.

### Growth rate assays

To examine the growth rates of *C. neoformans* on solid media, the wild type strain H99 was first grown for 2 days on YPD agar at 30°C. Cells were then replated on YPD or 10% YPD plates and cultured in air or 5% CO_2_ at 37°C for 7 days. Colonies were imaged at specific time points as indicated in the figures and colonies diameters were measured. Total colonies were collected and resuspended in 1mL PBS. Five µL of serial dilutions were dropped onto YPD plates and cultured for 2 days at 30°C. Colony-forming units (CFU) were counted to determine the cell number per colony. Four independent colonies were analyzed per group.

### Transmission electron microscopy

Transmission electron microscopy (TEM) was performed based on a previous report with slight modifications [62]. Cells collected from infected mouse brain tissues or from *in vitro* cultures were fixed with 5% glutaraldehyde for 4h at 4°C. The fixative solution was removed by washing three times with 0.1M PBS and cells were embedded in 1% low temperature gelling agarose. Cells were post fixed with 1% OsO_4_ in 0.1 M PBS (pH 7.4) for 2 h at room temperature followed by three washes in PBS for 15 minutes. Dehydration was performed using a graded concentration series of ethanol (30%, 50%, 60%, 70%, 80%, 95%,100%, and 100%), followed by two washes in 100% acetone. Cells were then embedded in resin, and 60–80 nm sections were cut using an ultramicrotome. Sections were placed on cuprum grids with formvar film and stained with 2% uranyl acetate (UA) for 8 minutes in the dark, washed three times with 70% ethanol and rinsed three times with ddH_2_O. Subsequently, sections were stained with 2.6% Reynolds’ Lead Citrate for 8 minutes and washed three times with ddH_2_O. After drying, the cuprum grids were observed using a Hitachi HT7800/HT7700 transmission electron microscope and images were acquired.

### RNA-seq analysis

*C. neoformans* H99 cells were cultured on 100% YPD or 10% YPD in the presence or absence of 5% CO_2_ for 5 days at 37 °C. Cells were collected and total RNA was extracted as described previously [57]. Two biological replicates were prepared for each sample. The quantity of RNA was assessed by measuring the OD at 260 nm and 280 nm using a Nanodrop-2000 Ultraviolet Spectrophotometer (Thermo Fisher, China). All samples had an A260: A280 ratio between 1.8 and 2.0. RNA integrity was evaluated using the Agilent 2200 Tape Station (Agilent Technologies, USA, RNA integrity number above 7.0). Libraries were sequenced on the Illumina NovaSeq platform according to the manufacturer’s instructions (performed by Berry Genomics Co., Beijing, China). Approximately 3 GB of data were obtained per library. Raw RNA-Seq data are available in the NCBI BioProject PRJNA1466205. Low-quality (Phred score<10), ambiguous, and adaptor bases were removed using FASTX-Toolkit v0.0.14 (http://hannonlab.cshl.edu/fastx_toolkit/index.html). Clean reads were aligned to the *C. neoformans* H99 reference genome using HiSat2 v2.0.5 with default parameters. Transcript expression was estimated with StringTie v1.3.3b using default parameters [63]. Differentially expressed genes were analyzed using R package DESeq2 [64]. Differentially expressed genes satisfy two criteria: (i) a fold change value higher than or equal to 2; (ii) an adjusted p-value (false discovery rate [FDR]) lower than 0.05. Heatmaps of differentially expressed genes and GO analysis were generated using the FungiExpresZ web tool (https://cparsania.shinyapps.io/FungiExpresZ/).

### Murine infection and virulence assays

Yeast strains were grown overnight at 30 °C, washed twice with PBS, and resuspended to a final concentration of 1 × 10^6^ cells/mL. Female C57BL/6 or BALB/c mice of 8–9 weeks old were intranasally infected with 10^5^ yeast cells per strain as described previously [28]. Over the course of the experiments, animals that appeared moribund or other endpoint symptoms were euthanized. Survival data were analyzed between groups using the log-rank test (*P* values < 0.05 were considered statistically significant). Brain, lung, and spleen tissues of mice at the endpoint were isolated and homogenized. The tissue suspensions were serially diluted and plated on YPD agar, and colonies were counted after 2 days of incubation at 30°C. Additionally, a subgroup of infected lungs was harvested at 3 days post-inoculation. Tissue homogenates were treated with ddH_2_O to lyse host cells, and *C. neoformans* cells were imaged under light microscopy. Body and capsule sizes of over 100 cells were measured.

For the intravenous injection model, 10^5^ yeast cells in 100 μL PBS were inoculated via tail vein injection [65]. Groups of 10 female BALB/c mice were injected with typical or ovoid cells and survival rate was recorded. Fungal burdens in the brain, lung, and spleen at the endpoint were quantified. To compare virulence between typical cells and ovoid cells, organs of infected mice were harvested at 1h, 6h, and 24h post injection and fungal burdens were examined.

For histopathological analysis, portions of the brain, lung, and spleen were fixed in 10% neutral-buffered formalin. Samples were embedded in paraffin wax, sectioned at 5 μm, and stained with hematoxylin and eosin (H&E) according to standard protocols.

### Minimal inhibitory concentration testing

The minimal inhibitory concentration (MIC) of fluconazole was performed using the E-test method. *C. neoformans* H99 cells were plated on 10% YP + 100% D or 10% YP + 10% D plates. E-test strips containing fluconazole were placed on the plates, which were incubated in the presence or absence of 5% CO_2_ at 37 °C. MIC values were read according to the manufacturer’s instructions. The disc diffusion method was used to test the susceptibility of fungal cells to fluconazole. 5,000 fungal cells collected from colonies with in the inhibition zone were plated on 10% YP + 100% D or 10% YP + 10% D plates. A paper disc was put on the middle of the plates and 5 μL of 50 mg/mL fluconazole were dropped onto the disc. Plates were incubated in 5% CO_2_ at 37 °C.

### Antifungal drug killing assay

Cells collected from the indicated conditions were resuspended in liquid medium as indicated and treated with 80 μg/mL fluconazole for 6 days. The relative tolerance rate was calculated as the ratio of colony forming units in fluconazole treated samples to untreated controls.

## Supporting information

S1 Fig Representative images of colonies and cells of *C. neoformans* grown on YPD agar plates at concentrations of 100%, 50%, 25%, 10%, 5%, 2%, 1%, 0.5%, and 0% for 5 days at 37°C with or without 5% CO_2_. Scale bar, 1mm.
(TIF)

S2 Fig Representative images of colonies and cells of *C. neoformans* grown on 100%, 50%, 25%, 10%, 5%, 2%, 1%, and 0.5% DMEM or RPMI1640 agar plates for 5 days at 37°C with or without 5% CO_2_. Scale bar for colony, 1mm, scale bar for colony, 5 µm.
(TIF)

S3 Fig Effects of pH on the formation of small cells in *C. neoformans.* Representative colonies and cells of *C. neoformans* grown on 10% and 100% YPD agar plates at different pH values for 5 days at 37°C. Scale bar for colony, 1mm, scale bar for cell, 5 µm.
(TIF)

S4 Fig *OSP1* is highly expressed in ovoid cells. (a) FPKM value of *OSP1* in cells grown on 10% YPD in 5% CO_2_, 10% YPD under normoxia, and 100% YPD in 5% CO_2_. Error bars indicate the standard error of the mean. Statistical analysis was done by a two-tailed t test comparing mutant and H99 strains. **, P < 0.01, ***, P < 0.001. (b) Representative images to show Osp1:mCherry on solid media. The mCherry tagged strain was cultured on the indicated agar plated as for 5 days. DIC, differential interference contrast. Scale bar, 5 µm. (c) Quantitative analysis the percentage of ovoid cells expressing *OSP1*. Percentage data are cumulative from four independent experiments and cell size data (n = 300 cells) are representative of four independent experiments. Error bars indicate the standard error of the mean. Statistical analysis was done by a two-tailed t test. ***, P < 0.001. (d) Representative images to show Osp1:mCherry in liquid media. The mCherry tagged strain was cultured in liquid media for 2 days. Scale bar, 5 µm.
(TIF)

S5 Fig Colony and cell morphology on different concentrations of YP plates at 37°C. Representative images of *C. neoformans* grown on 100%, 50%, 25%, 10%, 5%, 2%, 1%, and 0.5% YP for 5 days at 37°C. Scale bar for colony, 1mm, scale bar for cell, 5 µm.
(TIF)

S6 Fig Effects of pH on the formation of small cells in *C. neoformans.* Representative images of colonies and cells grown for 5 days on 10% YP + 10% D and 10% YP + 100% D agar at different pH values at 37°C with or without CO_2_. Scale bar for colony, 1mm, scale bar for cell, 5 µm.
(TIF)

S7 Fig Cell body size under different growth conditions. (a) Colony and cellular morphologies of *HXK2*, *MIG1*, or *PKA1* deletion mutants grown on 10% YP containing different concentrations of glucose for 5 days in air. Scale bar for colony, 1mm, scale bar for cell, 5 µm. (b) Quantitative measurement of cell body size from *HXK2*, *MIG1*, or *PKA1* deletion mutants and gene complemented strains grown on 10% YP with 10% or 100% D under normoxia. Data are compiled from 100 cells per group. Error bars indicate the 95% confidence interval of the median. **, P < 0.01, ***, P < 0.001.
(TIF)

S8 Fig Colony and cellular morphologies of *RIM101*, *CRK1*, *FBP1,* or *PHO4* deletion mutants grown on 100% YPD or 10% YPD for 5 days in 5% CO_2_. Scale bar for colony, 1mm, scale bar for cell, 5 µm.
(TIF)

S9 Fig Characteristics of ovoid cells. (a) Representative images to show Nop1 localization in cells grown on 100% YPD or 10% YPD in the presence or absence of CO_2_ (left). Quantitative measurement of the diameter ratio of nuclear to cell using Nop1:mCherry signal (right). Data are compiled from 100 cells per group. Error bars indicate the 95% confidence interval of the median. Statistical analysis was performed with Tukey’s multiple comparisons test. ***, P < 0.001. Scale bar, 5 µm. (b) DAPI staining of cells grown on 100% YPD or 10% YPD in the presence or absence of CO_2_ (left). Quantitative measurement of the diameter ratio of nuclear to cell using DAPI signal (right). Data are compiled from 100 cells per group. Error bars indicate the 95% confidence interval of the median. Statistical analysis was performed with Tukey’s multiple comparisons test. ***, P < 0.001. Scale bar, 5 µm. (c) CFW staining of seed cells and titanides. DIC, differential interference contrast, CFW, Calcoﬂuor white. Scale bars, 5 µm. (d) Representative images to show Nop1 localization in seed cells and titanides. Scale bars, 5 µm.
(TIF)

S10 Fig Representative colony images to show growth on 100% YPD or 10% YPD with or without CO_2_ at indicated time points. Scale bar, 1mm.
(TIF)

S11 Fig Virulence of ovoid cells in a murine inhalation model. (a) Survival curves of C57BL/6 mice after injection with cells grown under indicated conditions. (b) Fungal burden in organs of infected mice at the endpoint. Data are pooled from 9 mice per group. Error bars indicate the 95% confidence interval of the median. (c) Fungal burden in lungs at 1-, 6-, and 24-hours post-infection. Data are cumulated from 3-4 mice per group. Error bars indicate the standard error of the mean. (d) Representative images to show ovoid cells in the brain, lung and spleen of infected mice. Cells recovered from lungs at 3dpi or three organs at 23 dpi from both BALB/c and C57BL/6 mouse strains were examined. DIC, differential interference contrast. Scale bar, 10 µm. (e) H&E-stained lung sections from infected mice at 3 days post-infection or endpoint and visualized by light microscopy. Bar, 10 µm.
(TIF)

S1 Table Functional category of differentially expressed genes. (XLSX)

S2 Table Raw measurement data. (XLSX)

S3 Table Strains used in this study. (DOCX)

S4 Table Primers used in this study. (DOCX)
