# Supplementary figures and images for: Ovoid cell is an inducible small-sized morphotype that enhances proliferation and antifungal drug tolerance in the human fungal pathogen Cryptococcus neoformans
Source: PLoS Pathog. 2026 Jun 17;22(6):e1014302. doi: 10.1371/journal.ppat.1014302 (PMC13274837; doi:10.1371/journal.ppat.1014302)

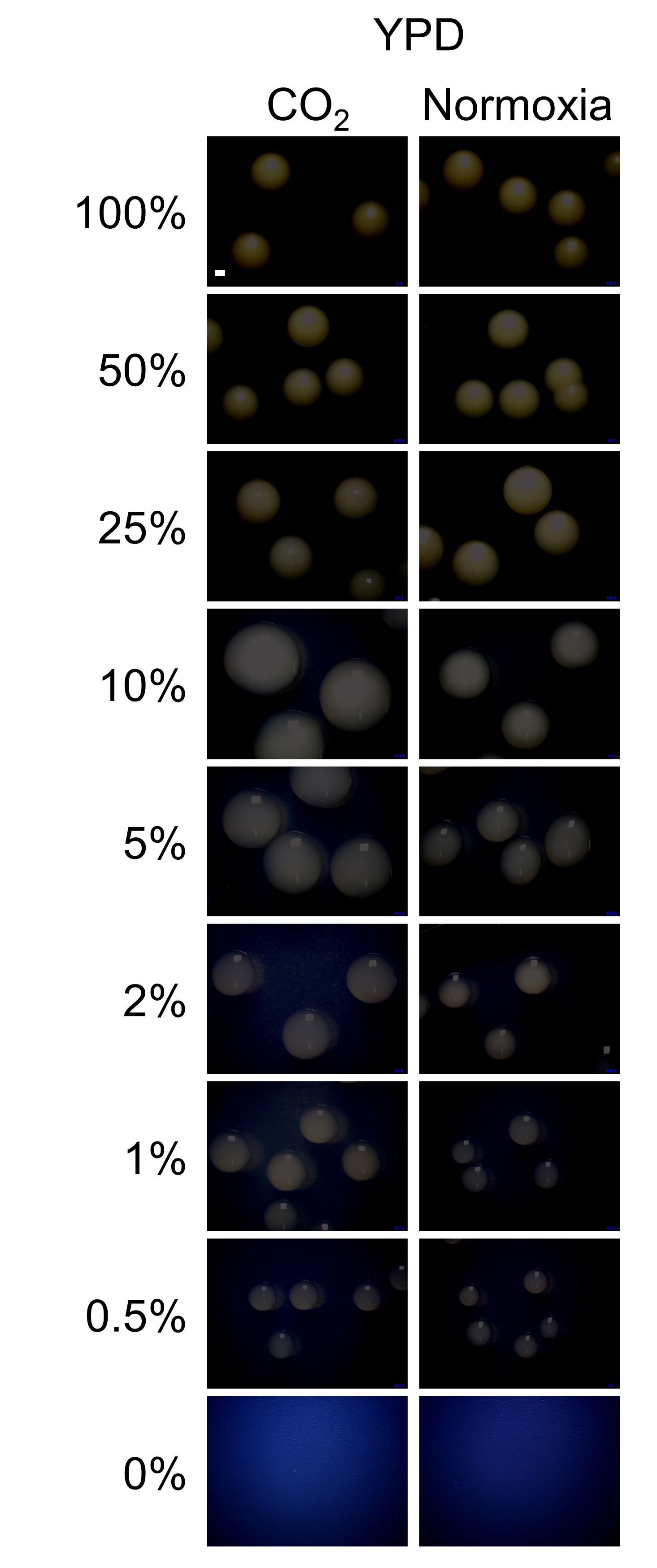

Supplement: S1 Fig — Scale bar, 1mm. (TIF) [file ppat.1014302.s001.tif]

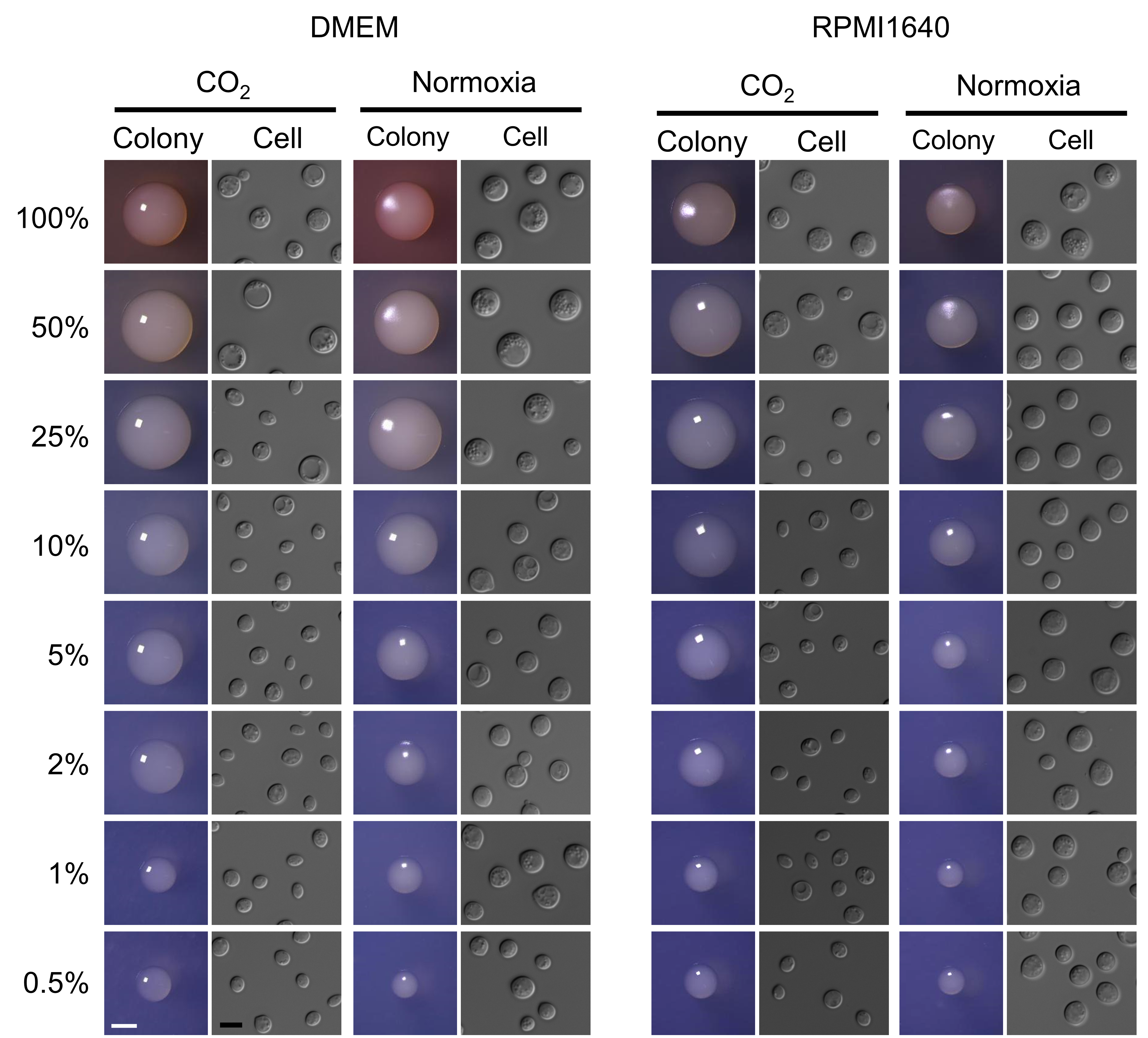

Supplement: S2 Fig — Scale bar for colony, 1mm, scale bar for colony, 5 µm. (TIF) [file ppat.1014302.s002.tif]

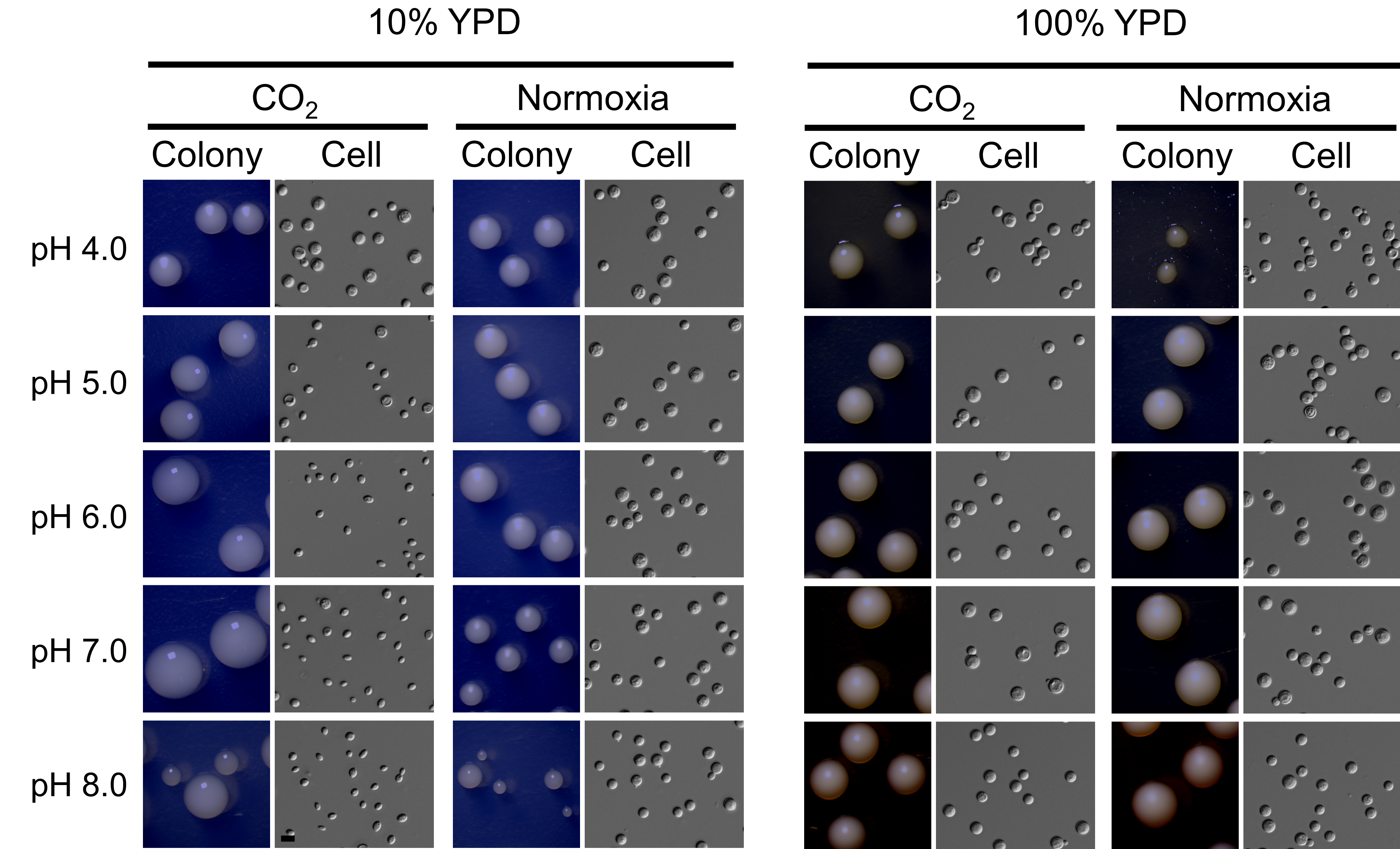

Supplement: S3 Fig — Representative colonies and cells of C. neoformans grown on 10% and 100% YPD agar plates at different pH values for 5 days at 37°C. Scale bar for colony, 1mm, scale bar for cell, 5 µm. (TIF) [file ppat.1014302.s003.tif]

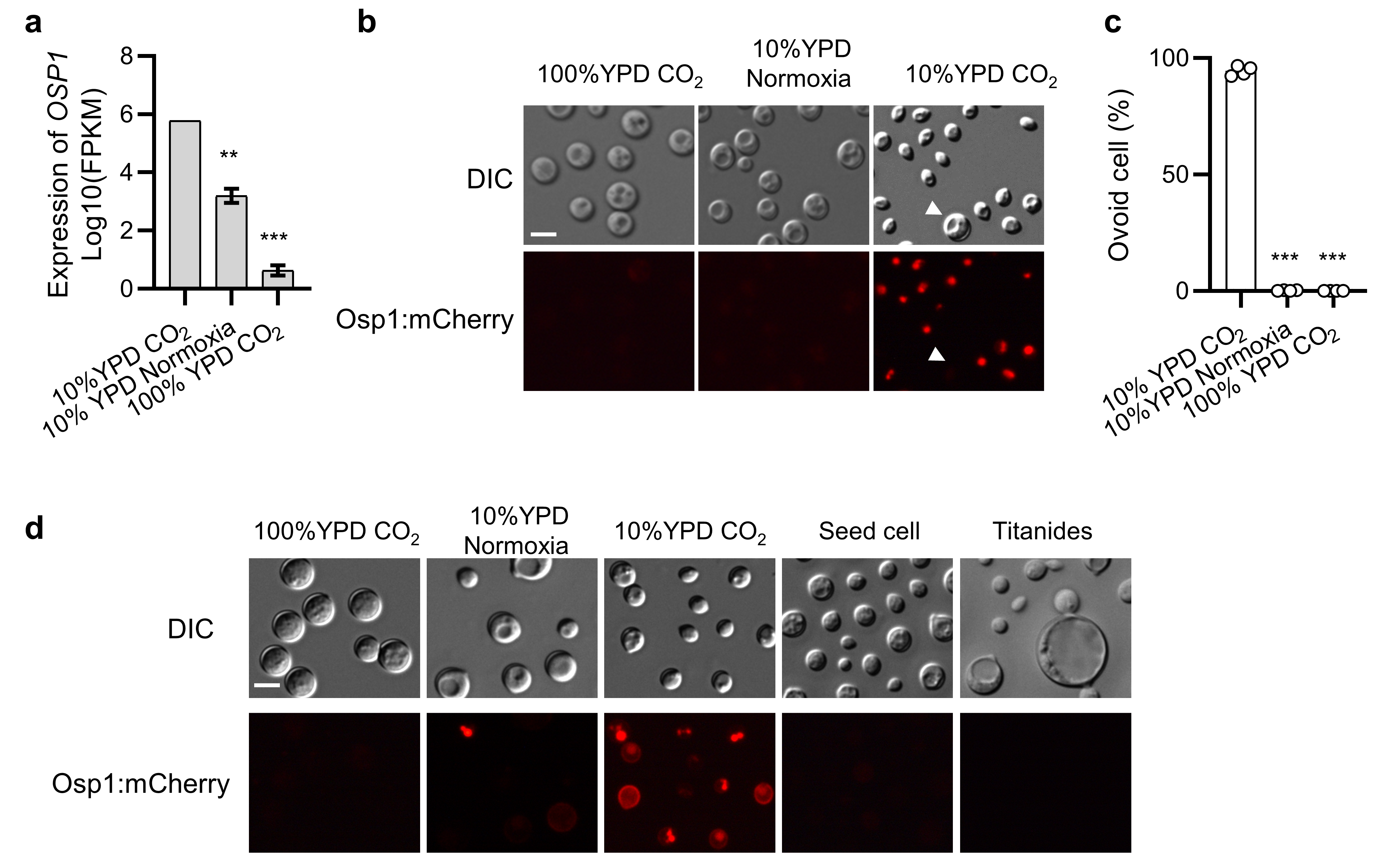

Supplement: S4 Fig — (a) FPKM value of OSP1 in cells grown on 10% YPD in 5% CO2, 10% YPD under normoxia, and 100% YPD in 5% CO2. Error bars indicate the standard error of the mean. Statistical analysis was done by a two-tailed t test comparing mutant and H99 strains. **, P < 0.01, ***, P < 0.001. (b) Representative images to show Osp1:mCherry on solid media. The mCherry tagged strain was cultured on the indicated agar plated as for 5 days. DIC, differential interference contrast. Scale bar, 5 µm. (c) Quantitative analysis the percentage of ovoid cells expressing OSP1. Percentage data are cumulative from four independent experiments and cell size data (n = 300 cells) are representative of four independent experiments. Error bars indicate the standard error of the mean. Statistical analysis was done by a two-tailed t test. ***, P < 0.001. (d) Representative images to show Osp1:mCherry in liquid media. The mCherry tagged strain was cultured in liquid media for 2 days. Scale bar, 5 µm. (TIF) [file ppat.1014302.s004.tif]

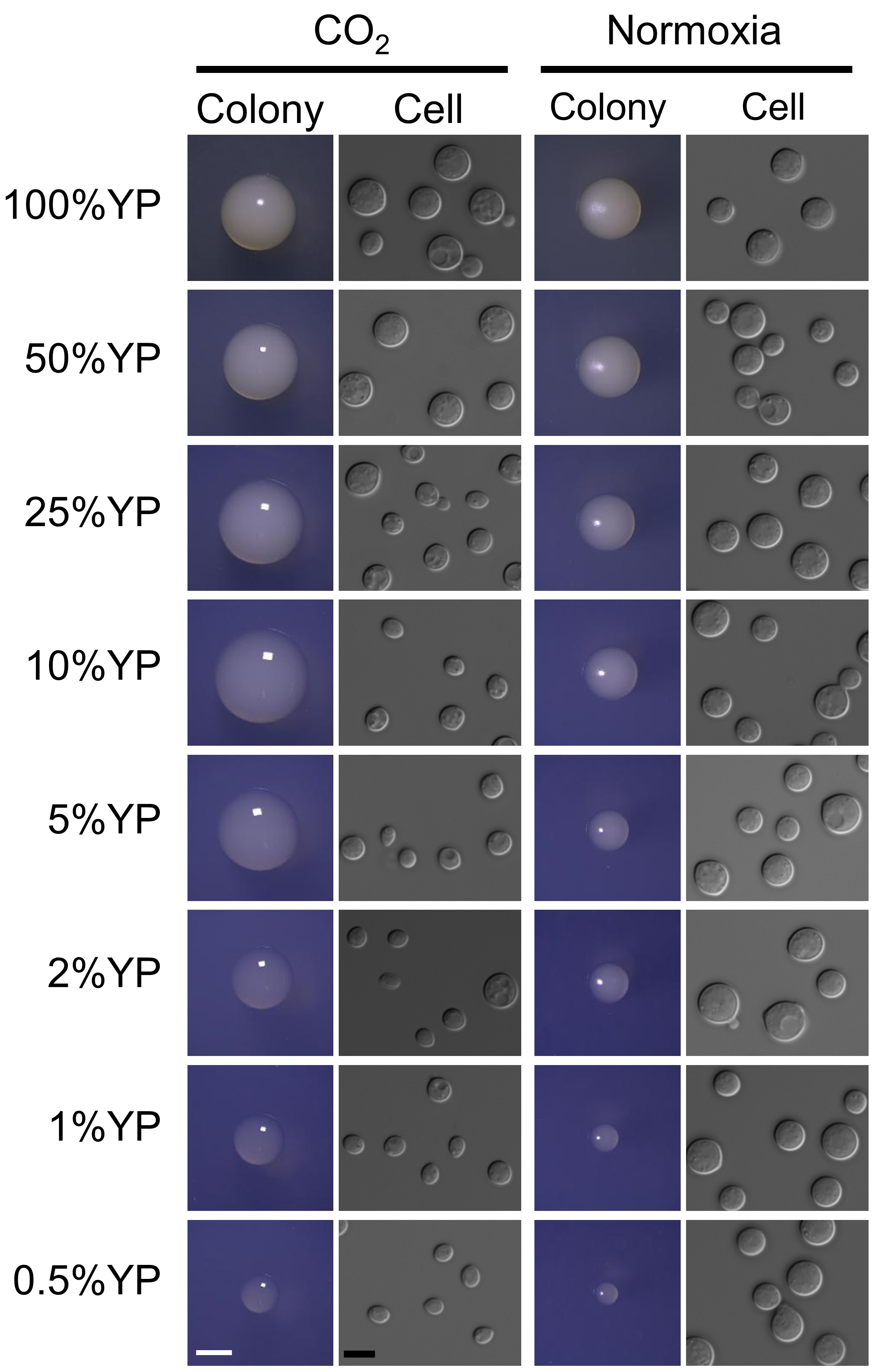

Supplement: S5 Fig — Representative images of C. neoformans grown on 100%, 50%, 25%, 10%, 5%, 2%, 1%, and 0.5% YP for 5 days at 37°C. Scale bar for colony, 1mm, scale bar for cell, 5 µm. (TIF) [file ppat.1014302.s005.tif]

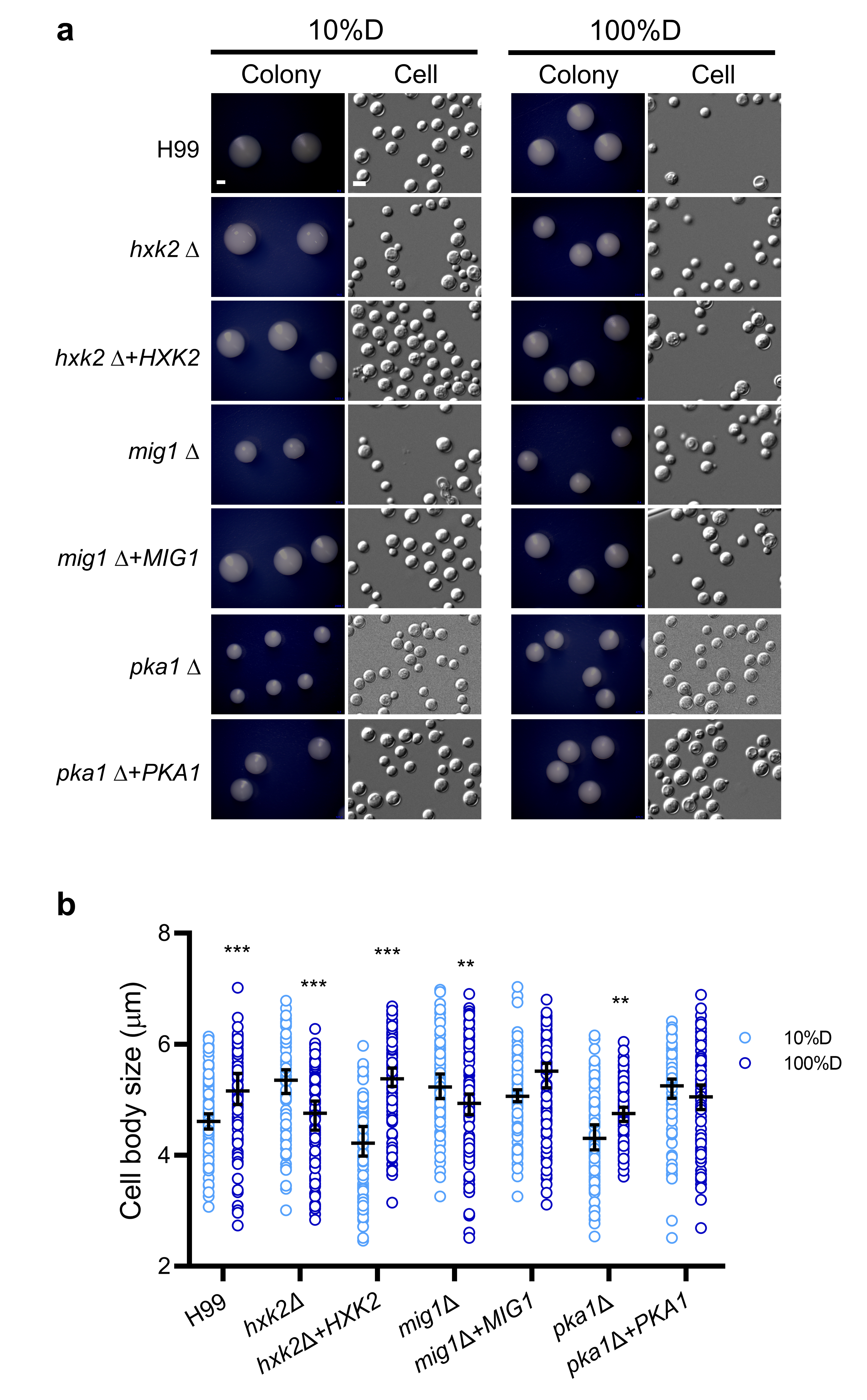

Supplement: S6 Fig — Representative images of colonies and cells grown for 5 days on 10% YP + 10% D and 10% YP + 100% D agar at different pH values at 37°C with or without CO2. Scale bar for colony, 1mm, scale bar for cell, 5 µm. (TIF) [file ppat.1014302.s006.tif]

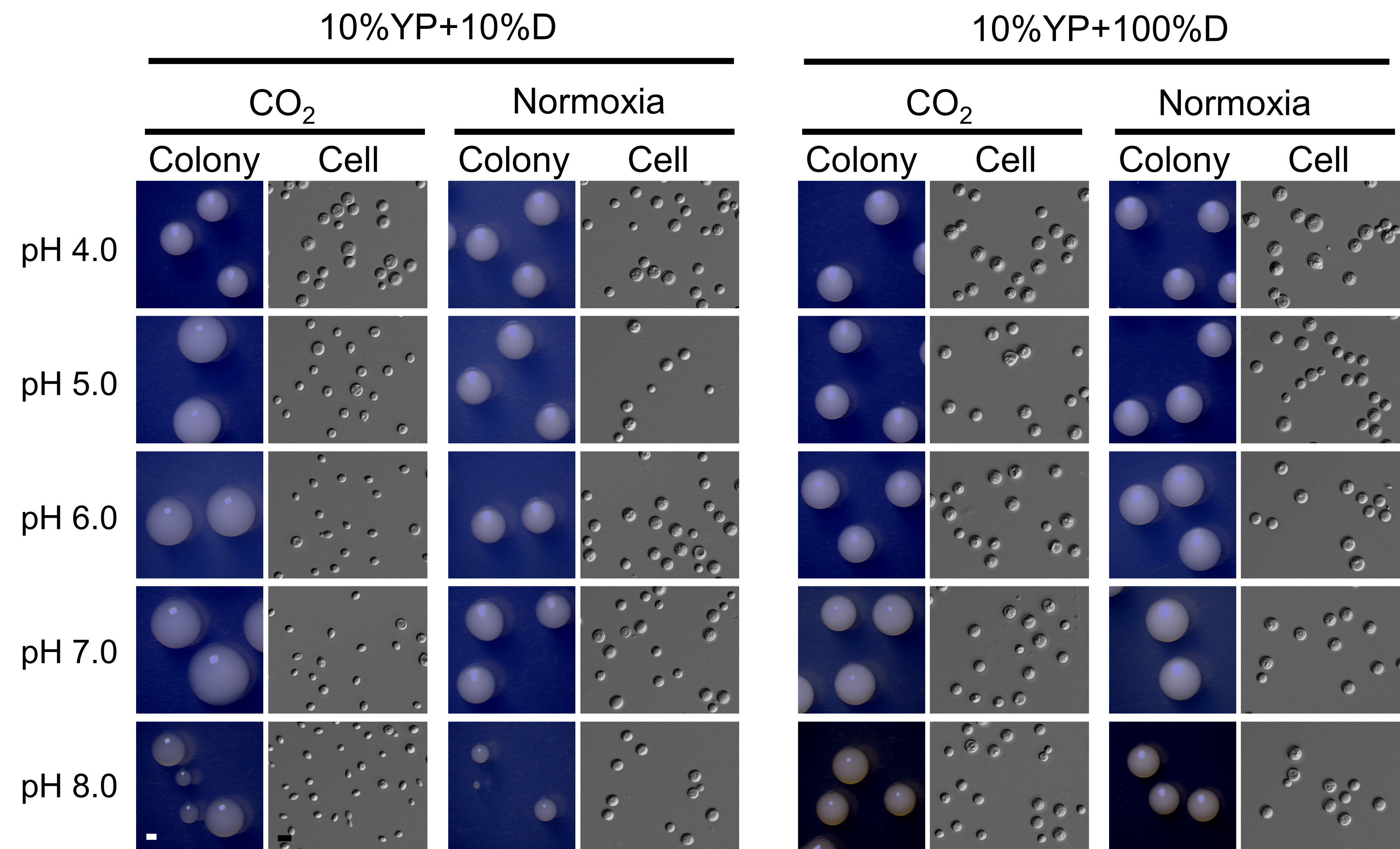

Supplement: S7 Fig — (a) Colony and cellular morphologies of HXK2, MIG1, or PKA1 deletion mutants grown on 10% YP containing different concentrations of glucose for 5 days in air. Scale bar for colony, 1mm, scale bar for cell, 5 µm. (b) Quantitative measurement of cell body size from HXK2, MIG1, or PKA1 deletion mutants and gene complemented strains grown on 10% YP with 10% or 100% D under normoxia. Data are compiled from 100 cells per group. Error bars indicate the 95% confidence interval of the median. **, P < 0.01, ***, P < 0.001. (TIF) [file ppat.1014302.s007.tif]

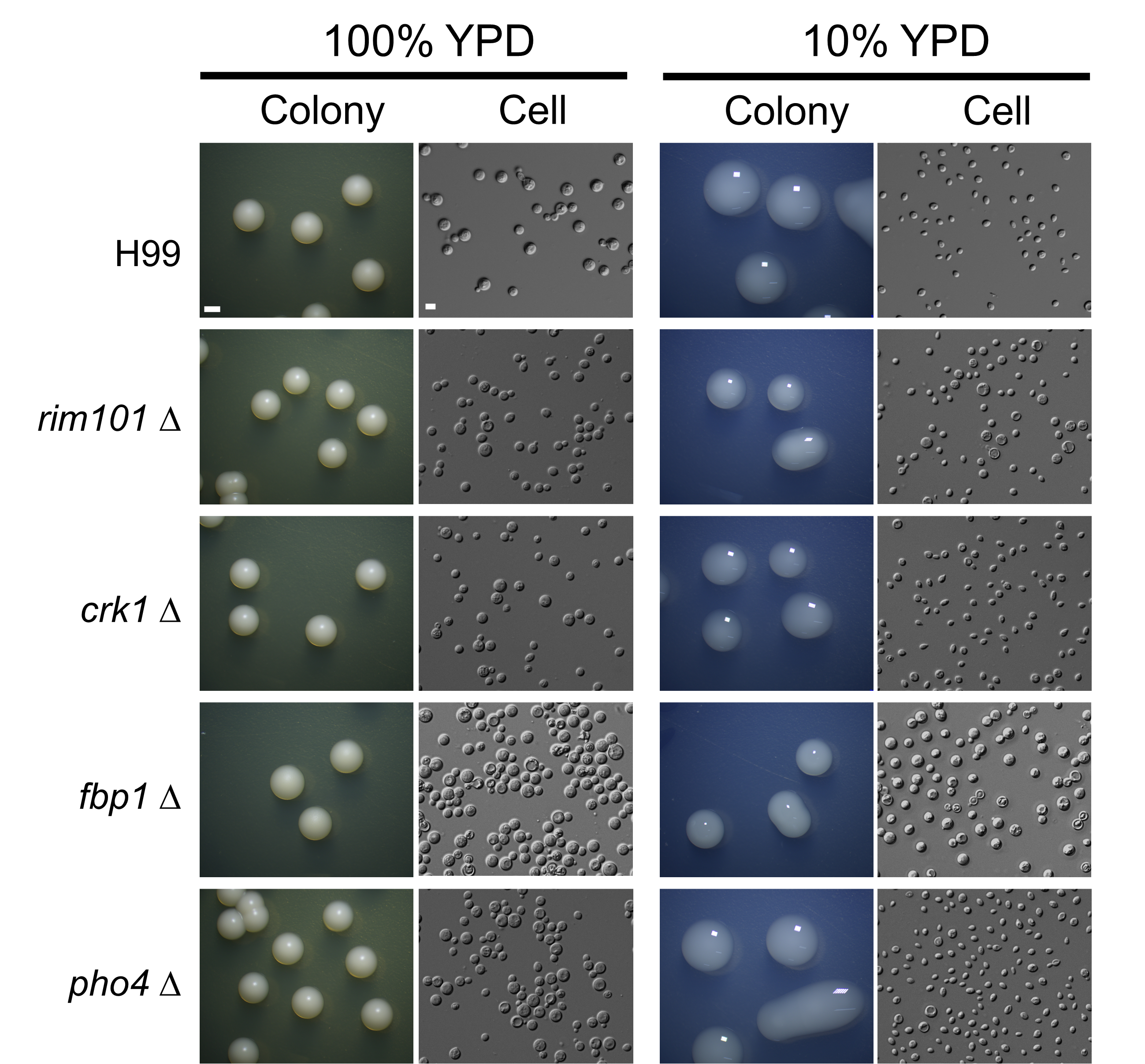

Supplement: S8 Fig — Scale bar for colony, 1mm, scale bar for cell, 5 µm. (TIF) [file ppat.1014302.s008.tif]

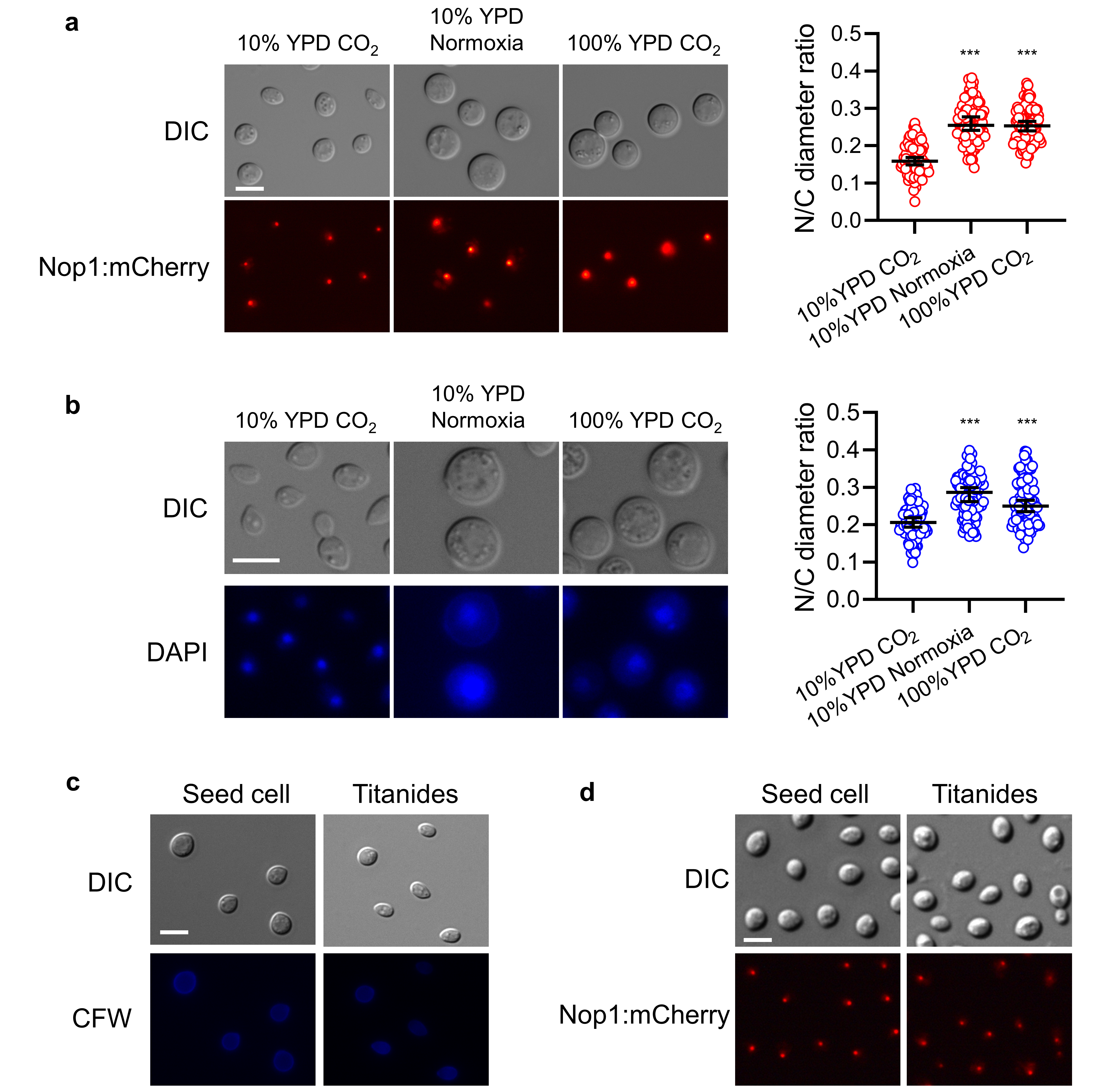

Supplement: S9 Fig — (a) Representative images to show Nop1 localization in cells grown on 100% YPD or 10% YPD in the presence or absence of CO2 (left). Quantitative measurement of the diameter ratio of nuclear to cell using Nop1:mCherry signal (right). Data are compiled from 100 cells per group. Error bars indicate the 95% confidence interval of the median. Statistical analysis was performed with Tukey’s multiple comparisons test. ***, P < 0.001. Scale bar, 5 µm. (b) DAPI staining of cells grown on 100% YPD or 10% YPD in the presence or absence of CO2 (left). Quantitative measurement of the diameter ratio of nuclear to cell using DAPI signal (right). Data are compiled from 100 cells per group. Error bars indicate the 95% confidence interval of the median. Statistical analysis was performed with Tukey’s multiple comparisons test. ***, P < 0.001. Scale bar, 5 µm. (c) CFW staining of seed cells and titanides. DIC, differential interference contrast, CFW, Calcoﬂuor white. Scale bars, 5 µm. (d) Representative images to show Nop1 localization in seed cells and titanides. Scale bars, 5 µm. (TIF) [file ppat.1014302.s009.tif]

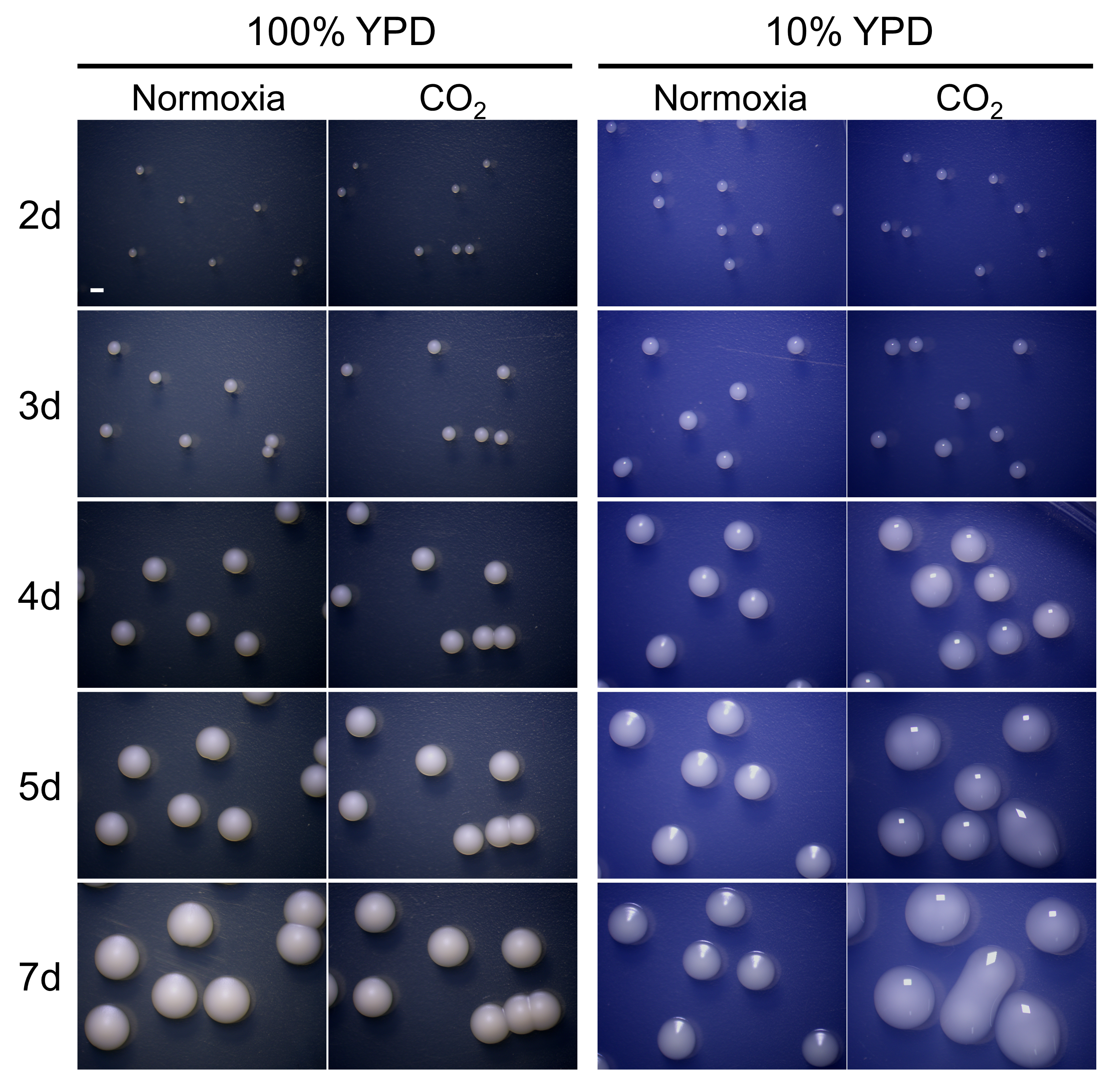

Supplement: S10 Fig — Scale bar, 1mm. (TIF) [file ppat.1014302.s010.tif]

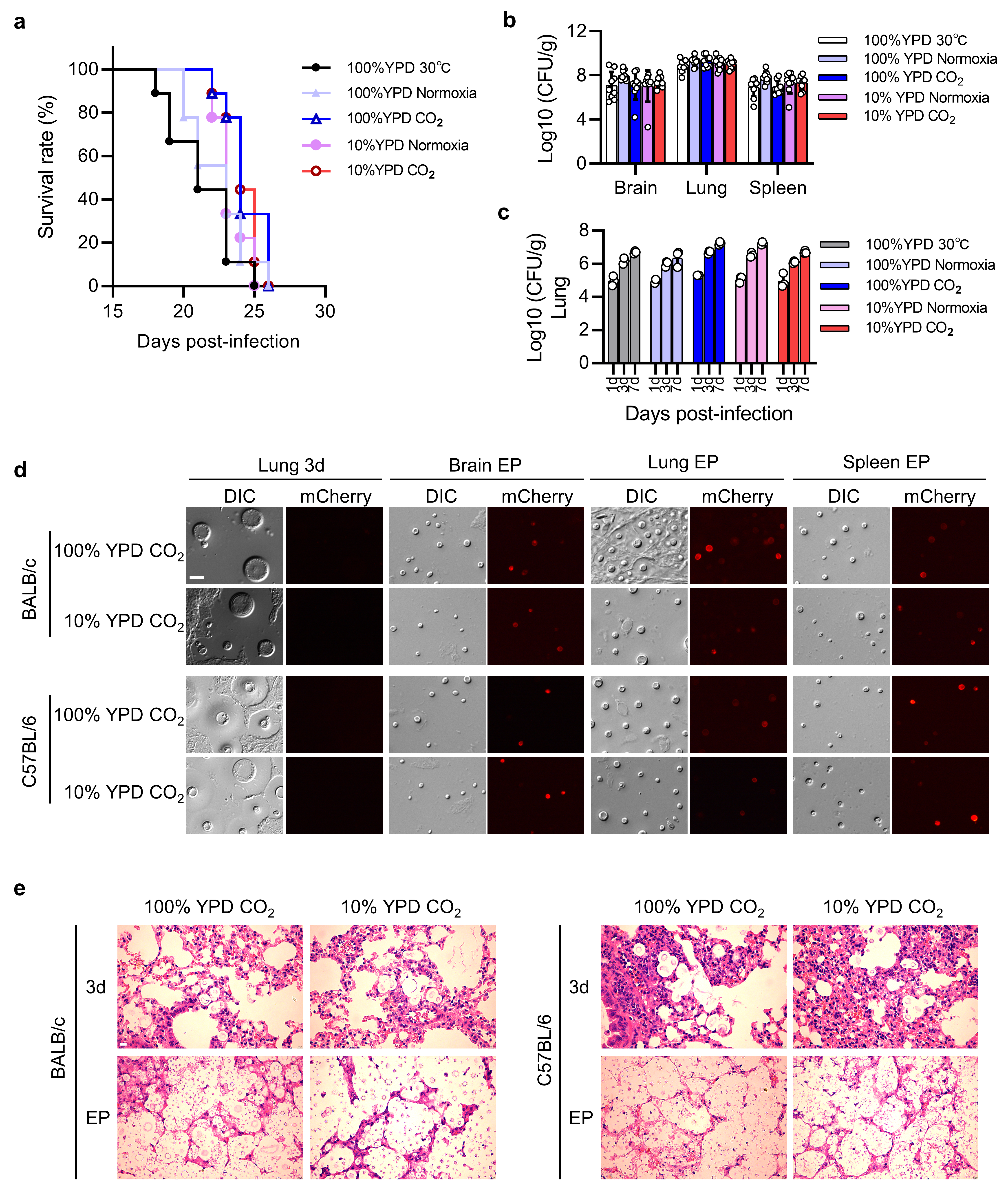

Supplement: S11 Fig — (a) Survival curves of C57BL/6 mice after injection with cells grown under indicated conditions. (b) Fungal burden in organs of infected mice at the endpoint. Data are pooled from 9 mice per group. Error bars indicate the 95% confidence interval of the median. (c) Fungal burden in lungs at 1-, 6-, and 24-hours post-infection. Data are cumulated from 3-4 mice per group. Error bars indicate the standard error of the mean. (d) Representative images to show ovoid cells in the brain, lung and spleen of infected mice. Cells recovered from lungs at 3dpi or three organs at 23 dpi from both BALB/c and C57BL/6 mouse strains were examined. DIC, differential interference contrast. Scale bar, 10 µm. (e) H&E-stained lung sections from infected mice at 3 days post-infection or endpoint and visualized by light microscopy. Bar, 10 µm. (TIF) [file ppat.1014302.s011.tif]
