# Supplementary material for: Ovoid cell is an inducible small-sized morphotype that enhances proliferation and antifungal drug tolerance in the human fungal pathogen Cryptococcus neoformans
Source: PLoS Pathog. 2026 Jun 17;22(6):e1014302. doi: 10.1371/journal.ppat.1014302 (PMC13274837; doi:10.1371/journal.ppat.1014302)
Supplement: S3 Table — (DOCX) [file ppat.1014302.s014.docx]

Table S3 Strains used in this study.

| Strains | Genotype | Species | Source |
| --- | --- | --- | --- |
| H99 | MATalpha | *Cryptococcus neoformans* | [1] |
| CCC608 | MATalpha *PACT1-Nop1-mCherry-NAT* | *C. neoformans* | [2] |
| CCC708 | MATalpha *hxk2::NAT* | *C. neoformans* | This study |
| CCC2025 | MATalph*a hxk2::NAT HXK2::NEO* | *C. neoformans* | This study |
| CCC1245 | MATalpha *mig1::NAT* | *C. neoformans* | This study |
| CCC1379 | MATalpha *mig1::NAT MIG1::NEO* | *C. neoformans* | This study |
| CCC716 | MATalph*a pka1::NAT* | *C. neoformans* | This study |
| CCC2031 | MATalpha *pka1::NAT PKA1::NEO* | *C. neoformans* | This study |
| CCC1056 | MATalpha *rim101::NAT* | *C. neoformans* | This study |
| *crk1*Δ | MATalpha *crk1::NEO* | *C. neoformans* | [3] |
| *fbp1*Δ | MATalpha *fbp1::NEO* | *C. neoformans* | [3] |
| *pho4*Δ | MATalpha *pho4::NAT* | *C. neoformans* | [4] |
| KN99 alpha | MATalpha | *C. neoformans* | [5] |
| XL280 | MATalpha | *C. neoformans* | [6] |
| AI187 | MATa/alpha diploid | *C. neoformans* | [7] |
| R265 | Clinical isolate, MATalpha VGII | *Cryptococcus gattii* | [8] |
| WM276 | Australian isolate MATalpha VGI | *C. gattii* | [8] |
| E566 | Australian isolate, MATa VGI | *C. gattii* | [9] |
| SC5314 | Clinical isolate, MTLa/alpha | *Candida albicans* | [10] |
| BJCA001 | Clinical isolate, MTLa | *Candida auris* | [11] |
| GH1374 | Clinical isolate, *MTL***a**/**a** | *Candida tropicalis* | [12] |
| JX1092 | Clinical isolate | *Candida glabrata* | [13] |
| ATCC6258 | Clinical isolate | *Candida krusei* | [13] |
| ATCC22019 | Clinical isolate | *Candida parapsilosis* | [13] |
| RJ1091 | Clinical isolate | *Candida dubliniensis* | [14] |

**Reference**

1. Perfect JR, Schell WA, Rinaldi MG. Uncommon invasive fungal pathogens in the acquired immunodeficiency syndrome. J Med Vet Mycol. 1993;31(2):175-9. PubMed PMID: 8509954.

2. Fan CL, Han LT, Jiang ST, Chang AN, Zhou ZY, Liu TB. The Cys(2)His(2) zinc finger protein Zfp1 regulates sexual reproduction and virulence in *Cryptococcus neoformans*. Fungal Genet Biol. 2019;124:59-72. Epub 2019/01/11. doi: 10.1016/j.fgb.2019.01.002. PubMed PMID: 30630094.

3. Cao C, Wang K, Wang Y, Liu TB, Rivera A, Xue C. Ubiquitin proteolysis of a CDK-related kinase regulates titan cell formation and virulence in the fungal pathogen *Cryptococcus neoformans*. Nat Commun. 2022;13(1):6397. Epub 2022/10/28. doi: 10.1038/s41467-022-34151-6. PubMed PMID: 36302775; PubMed Central PMCID: PMCPMC9613880.

4. Jung KW, Yang DH, Maeng S, Lee KT, So YS, Hong J, et al. Systematic functional profiling of transcription factor networks in Cryptococcus neoformans. Nat Commun. 2015;6:6757. doi: 10.1038/ncomms7757. PubMed PMID: 25849373; PubMed Central PMCID: PMCPMC4391232.

5. Nielsen K, Cox GM, Wang P, Toffaletti DL, Perfect JR, Heitman J. Sexual cycle of *Cryptococcus neoformans* var. *grubii* and virulence of congenic a and alpha isolates. Infect Immun. 2003;71(9):4831-41. doi: 10.1128/iai.71.9.4831-4841.2003. PubMed PMID: 12933823; PubMed Central PMCID: PMCPMC187335.

6. Zhai B, Zhu P, Foyle D, Upadhyay S, Idnurm A, Lin X. Congenic strains of the filamentous form of *Cryptococcus neoformans* for studies of fungal morphogenesis and virulence. Infect Immun. 2013;81(7):2626-37. Epub 2013/05/15. doi: 10.1128/IAI.00259-13. PubMed PMID: 23670559; PubMed Central PMCID: PMCPMC3697605.

7. Ianiri G, Idnurm A. Essential gene discovery in the basidiomycete *Cryptococcus neoformans* for antifungal drug target prioritization. Mbio. 2015;6(2). doi: 10.1128/mBio.02334-14. PubMed PMID: 25827419; PubMed Central PMCID: PMCPMC4453551.

8. Kidd SE, Hagen F, Tscharke RL, Huynh M, Bartlett KH, Fyfe M, et al. A rare genotype of *Cryptococcus gattii* caused the cryptococcosis outbreak on Vancouver Island (British Columbia, Canada). Proc Natl Acad Sci U S A. 2004;101(49):17258-63. Epub 2004/12/02. doi: 10.1073/pnas.0402981101. PubMed PMID: 15572442; PubMed Central PMCID: PMCPMC535360.

9. Krockenberger MB, Malik R, Ngamskulrungroj P, Trilles L, Escandon P, Dowd S, et al. Pathogenesis of pulmonary *Cryptococcus gattii* infection: a rat model. Mycopathologia. 2010;170(5):315-30. Epub 2010/06/17. doi: 10.1007/s11046-010-9328-z. PubMed PMID: 20552280.

10. Fonzi WA, Irwin MY. Isogenic strain construction and gene mapping in *Candida albicans*. Genetics. 1993;134(3):717-28. Epub 1993/07/01. doi: 10.1093/genetics/134.3.717. PubMed PMID: 8349105; PubMed Central PMCID: PMCPMC1205510.

11. Wang X, Bing J, Zheng Q, Zhang F, Liu J, Yue H, et al. The first isolate of *Candida auris* in China: clinical and biological aspects. Emerg Microbes Infect. 2018;7(1):93. Epub 2018/05/20. doi: 10.1038/s41426-018-0095-0. PubMed PMID: 29777096; PubMed Central PMCID: PMCPMC5959928.

12. Zhang Q, Tao L, Guan G, Yue H, Liang W, Cao C, et al. Regulation of filamentation in the human fungal pathogen *Candida tropicalis*. Mol Microbiol. 2016;99(3):528-45. Epub 2015/10/16. doi: 10.1111/mmi.13247. PubMed PMID: 26466925.

13. Zheng Q, Liu J, Qin J, Wang B, Bing J, Du H, et al. Ploidy variation and spontaneous haploid-diploid switching of *Candida glabrata* clinical isolates. mSphere. 2022;7(4):e0026022. Epub 2022/06/22. doi: 10.1128/msphere.00260-22. PubMed PMID: 35727043; PubMed Central PMCID: PMCPMC9429935.

14. Yue H, Hu J, Guan G, Tao L, Du H, Li H, et al. Discovery of the gray phenotype and white-gray-opaque tristable phenotypic transitions in *Candida dubliniensis*. Virulence. 2016;7(3):230-42. Epub 2015/12/30. doi: 10.1080/21505594.2015.1135287. PubMed PMID: 26714067; PubMed Central PMCID: PMCPMC4871672.l PMCID: PMCPMC4871672.
