# Supplementary material for: Ovoid cell is an inducible small-sized morphotype that enhances proliferation and antifungal drug tolerance in the human fungal pathogen Cryptococcus neoformans
Source: PLoS Pathog. 2026 Jun 17;22(6):e1014302. doi: 10.1371/journal.ppat.1014302 (PMC13274837; doi:10.1371/journal.ppat.1014302)
Supplement: S4 Table — (DOCX) [file ppat.1014302.s015.docx]

Table S4 Primers used in this study.

| Primers | Sequences (5’-3’) | Description |
| --- | --- | --- |
| M13F | GTAAAACGACGGCCAG | Marker amplify F |
| M13R | CAGGAAACAGCTATGAC | Marker amplify R |
| Selective F | TGCCCCTAAGAATTCGTG | Positive screening F |
| Selective R | TGGCGGAGGATAGAAGCTG | Positive screening R |
| *HXK2* F1 | CGGTATCGATAAGCTTTCTCTCCTTCACAGCTGG | *HXK2* KO F1 |
| *HXK2* R1 | GCACTGGCCGTCGTTTTACTGTGTGGCAGTCTGTAAG | *HXK2* KO R1 |
| *HXK2* F2 | CATGTCATAGCTGTTTCCTGACTGTGGCCTAATTCGATAG | *HXK2* KO F2 |
| *HXK2* R2 | ATTCGATATCAAGCTTAGTTGATATAGGAGACGC | *HXK2* KO R2 |
| *HXK2* F3 | TGTAGCTCATCTCAACTGTC | *HXK2* KO F3 |
| *HXK2* R3 | TCCATGTAAGTAGTTTGCC | *HXK2* KO R3 |
| *HXK2* F4 | AGACTCACTGTACCTTTACCTG | *HXK2* KO F4 |
| *HXK2* R4 | TCGGCTGTCTCTTGAATG | *HXK2* KO R4 |
| *HXK2*_sgRNA_U6_R | GCTGTTGGAGGGCCTTATCGAACAGTATACCCTGCCGGTG | *HXK2* KO sgRNA F |
| *HXK2*_sgRNA_gRNA_F | CCGCCATCAATGCTGCGGCTACTGTTTTAGAGCTAGAAATAGCAAGTT | *HXK2* KO sgRNA R |
| *HXK2* comp F | GCAGCCCGGGGGATCCTCACTGCGTTCTCTCCTTC | *HXK2* comp F |
| *HXK2* comp R | TGCTCACCATGGATCCAGGTCCACGTTCACCAGG | *HXK2* comp R |
| *PKA1* F1 | CGGTATCGATAAGCTTTCGATTGCCATATGTCATG | *PKA1* KO F1 |
| *PKA1* R1 | GCACTGGCCGTCGTTTTACACGCTACTGTATCGGATTG | *PKA1* KO R1 |
| *PKA1* F2 | CATGTCATAGCTGTTTCCTGTCTAATATGTGCTGCTGATC | *PKA1* KO F2 |
| *PKA1* R2 | ATTCGATATCAAGCTTTGAGATGTGCTGCAGTTG | *PKA1* KO R2 |
| *PKA1* F3 | ACGTATATCAAGCTAACACC | *PKA1* KO F3 |
| *PKA1* R3 | TCGCCAAGTATGTACCTG | *PKA1* KO R3 |
| *PKA1* F4 | TATCTCTAGCCATTTTCC | *PKA1* KO F4 |
| *PKA1* R4 | TGGGTCCTATCACATACG | *PKA1* KO R4 |
| *PKA1*_sgRNA_U6_R | AGTAGCCGCAGCATTGATGGAACAGTATACCCTGCCGGTG | *PKA1* KO sgRNA F |
| *PKA1*_sgRNA_gRNA_F | CCATCAATGCTGCGGCTACTGTTTTAGAGCTAGAAATAGCAAGTT | *PKA1* KO sgRNA R |
| *PKA1* comp F | GCAGCCCGGGGGATCCAGATTTCGGATTCTTGGC | *PKA1* comp F |
| *PKA1* comp R | TGCTCACCATGGATCCAAACTCCACGAAGAAATGAT | *PKA1* comp R |
| *MIG1* F1 | CGGTATCGATAAGCTTTCATACTCTCGTGCACCAG | *MIG1* KO F1 |
| *MIG1* R1 | GCACTGGCCGTCGTTTTACAAGGTTGCTATTGGATCTG | *MIG1* KO R1 |
| *MIG1* F2 | CATGTCATAGCTGTTTCCTGTAGCGTGATGAGTTTGATG | *MIG1* KO F2 |
| *MIG1* R2 | ATTCGATATCAAGCTTAGATGGATTTGGGATACTTG | *MIG1* KO R2 |
| *MIG1* F3 | TGTCCTCTATGTGATCGG | *MIG1* KO F3 |
| *MIG1* R3 | ACCACCAAATCCACTAGAG | *MIG1* KO R3 |
| *MIG1* F4 | TACCAACAACAATCGACAC | *MIG1* KO F4 |
| *MIG1* R4 | TGGGTCCTATCACATACG | *MIG1* KO R4 |
| *MIG1*_sgRNA_U6_R | TTGCTCTTCCTGGCACCAGCAACAGTATACCCTGCCGGTG | *MIG1* KO sgRNA F |
| *MIG1*_sgRNA_gRNA_F | GCTGGTGCCAGGAAGAGCAAGTTTTAGAGCTAGAAATAGCAAGTT | *MIG1* KO sgRNA R |
| *MIG1* comp F | GCAGCCCGGGGGATCCACCCTCCTGCTGACGAAG | *MIG1* comp F |
| *MIG1* comp R | TGCTCACCATGGATCCCGGATGTCCATCCAGCTC | *MIG1* comp R |
| *RIM101* F1 | ATCATCAGTAGTGCATGGC | *RIM101* KO F1 |
| *RIM101* R1 | GCACTGGCCGTCGTTTTACTCGAGTGTGGGAAAAAGTG | *RIM101* KO R1 |
| *RIM101* F2 | CATGTCATAGCTGTTTCCTGAATTCTTGCTCGGTGTGC | *RIM101* KO F2 |
| *RIM101* R2 | TGAATGCAGGGTTTATGATG | *RIM101* KO R2 |
| *RIM101* F3 | AGTACTTCATCCCACAGCC | *RIM101* KO F3 |
| *RIM101* R3 | AATTGAGACCGTACATCCC | *RIM101* KO R3 |
| *RIM101* F4 | TCTCATTAACCTCATGTTGC | *RIM101* KO F4 |
| *RIM101* R4 | TCCTTCTCCAATCACTCG | *RIM101* KO R4 |
| *RIM101*_sgRNA_U6_R | CATGGGGTGAGGTCCAGCTTAACAGTATACCCTGCCGGTG | *RIM101* KO sgRNA F |
| *RIM101*_sgRNA_gRNA_F | AAGCTGGACCTCACCCCATGGTTTTAGAGCTAGAAATAGCAAGTT | *RIM101* KO sgRNA R |
| Pnativ*e-OSP1* F | GCAGCCCGGGGGATCCACCTCATTATAGACCCGACG | *OSP1* comp F |
| Pnativ*e-OSP1* R | TGCTCACCATGGATCCATCAACATTTGCAGGGTTGT | *OSP1* comp R |
